# Single-Objective Lattice Light Sheet Microscopy with Microfluidics for Single-Molecule Super-Resolution Imaging of Mammalian Cells

**DOI:** 10.1021/acsphotonics.5c02201

**Published:** 2025-12-12

**Authors:** Siyang Cheng, Nahima Saliba, Gabriella Gagliano, Prakash Joshi, Anna-Karin Gustavsson

**Affiliations:** † Department of Chemistry, 3990 Rice University, Houston, Texas 77005, United States; ‡ Smalley-Curl Institute, Rice University, Houston, Texas 77005, United States; § Applied Physics Program, Rice University, Houston, Texas 77005, United States; ∥ Department of BioSciences, Rice University, Houston, Texas 77005, United States; ⊥ Department of Electrical and Computer Engineering, Rice University, Houston, Texas 77005, United States; # Center for Nanoscale Imaging Sciences, Rice University, Houston, Texas 77005, United States; ∇ Department of Cancer Biology, University of Texas MD Anderson Cancer Center, Houston, Texas 77005, United States

**Keywords:** lattice light sheet, light sheet microscopy, single-objective, single-molecule localization microscopy, super-resolution imaging, microfluidics

## Abstract

Single-molecule localization microscopy (SMLM) has redefined optical imaging by enabling imaging beyond the diffraction limit, allowing for nanoscale investigation into cellular architecture and molecular dynamics. Light sheet illumination enhances SMLM through optical sectioning of the sample, which drastically improves the signal-to-background ratio and reduces photobleaching and photodamage. Lattice light sheet (LLS) microscopy, in which a 2D optical lattice is implemented for light sheet illumination, can provide exceptional sectioning and extended imaging depth when imaging in scattering samples. However, its conventional dual-objective design poses challenges for certain applications. Here, we present an imaging platform that implements LLS illumination with a reflective single-objective geometry (soLLS) inside a microfluidic chip, enabling the use of a single high numerical aperture objective for both illumination and detection, mitigating constraints of a dual-objective setup. We provide a quantitative characterization of the propagation properties of the soLLS and demonstrate that it outperforms conventional Gaussian light sheets in terms of useful field of view and sectioning when propagating through scattering samples. Next, by combining soLLS with point spread function engineering, we demonstrate the platform for improved 3D single-molecule super-resolution imaging of multiple targets across multiple cells. The soLLS imaging platform thus expands investigations of nanoscale cellular and intercellular structures and mechanisms into more challenging samples for a wide range of applications in biology and biomedicine.

## Graphical abstract

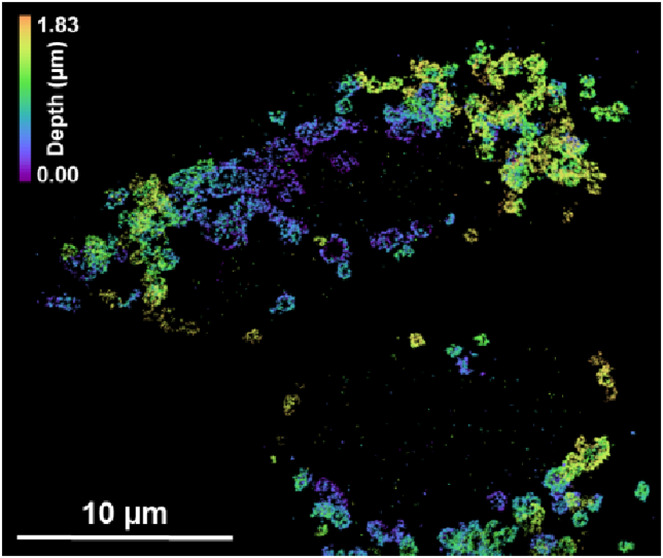

## Introduction

Fluorescence microscopy is an essential tool for investigating cellular and molecular processes, enabling specific and gentle visualization of subcellular structures.^1,2^ Expanding on this capacity, single-molecule localization microscopy (SMLM) has revolutionized the study of biological systems by temporally separating the emission of individual fluorophores and localizing them across sequential imaging frames with nanoscale precision, enabling nanoscale examination of cellular architectures and molecular dynamics beyond the optical diffraction limit.^3–6^ DNA points accumulation for imaging in nanoscale topography (DNA-PAINT) is one SMLM approach that utilizes transient binding of short dye-labeled oligonucleotide “imager” strands to their complementary “docking” strands on the target to generate temporally separated fluorescence signals for SMLM.^7,8^ However, the commonly employed wide-field epi-illumination strategy excites the entire sample simultaneously, resulting in a reduced signal-to-background ratio (SBR) due to high fluorescence background from out-of-focus emitters and increased risk of photobleaching and photodamage of the sample. In SMLM, the reduced SBR deteriorates the localization precision, which, in turn, degrades the overall resolution of the super-resolved reconstruction. Light sheet (LS) illumination, a strategy where a sheet of light is used to optically section the sample and restrict the illumination to the focal plane, can improve the SMLM performance by increasing the SBR and reducing the photobleaching and photodamage, while still allowing for simultaneous illumination of the entire field of view (FOV).^9–12^ Lattice light sheet (LLS) microscopy, which implements a 2D optical lattice for LS illumination, can provide superior penetration depth in thick and scattering samples.^13^ In LLS microscopy, the inherent trade-off between optical sectioning and useful FOV of conventional light sheets with a Gaussian beam profile (Gaussian LS) is mitigated by the nature of the optical lattice.^13,14^ Compared to Bessel beam illumination,^15^ a lattice light sheet (LLS) suppresses the side lobes of the Bessel beam, thereby reducing background fluorescence excited outside the image plane. Moreover, LLS microscopy reduces photobleaching and phototoxicity, making it more optimized for live-cell imaging applications.^13^ In combination with SMLM approaches such as DNA-PAINT^7,8^ and direct stochastic optical reconstruction microscopy (dSTORM),^16^ LLS microscopy has successfully been used for super-resolution imaging of cellular structures.^17^ It has also been demonstrated for dynamic imaging of living systems^13^ and for single-particle tracking of plasma membrane molecules.^18^

While widely adopted,^13,17,19,20^ the conventional two-objective design of LLS microscopy suffers from several drawbacks. First, the excitation and detection objectives are arranged in an orthogonal geometry, which limits the implementation of high numerical aperture (NA) and short working distance objectives due to physical constraints.^13,21^ Second, the objectives are dipped in a media-filled bath, increasing the risk of sample contamination. Third, microfluidics, which allows for automated and reversible solution exchange and control of the extracellular environment,^22–26^ is difficult to implement in two-objective LS setups. Although previous studies have demonstrated such integration, these approaches often present limitations, including limited objective lens selections,^27^ the need for special sample holders,^27,28^ the need for refractive index-matching films,^27,29,30^ or increased operational complexity compared to inverted microscopes due to strict incident angle requirement.^29^ Additionally, the geometry of dual-objective LLS imaging systems can introduce reconstruction artifacts, which require computational correction through postprocessing.^31^

In this work, we present a single-objective LLS (soLLS) imaging platform that alleviates drawbacks of the conventional two-objective LLS design. In soLLS, LLS illumination and fluorescence detection are achieved through the same high-NA objective lens, which enables the generation of a thin, high-quality, and well-defined LLS while also maintaining a high collection efficiency of photons emitted from the sample. To enable single-objective LLS illumination, the optical platform was inspired by the geometries of single-objective selective-plane illumination microscopy (soSPIM)^32^ and our recent work (soTILT3D),^33^ which both utilize conventional Gaussian LSs that are reflected into the sample. By using a reflective micro-optics inside a microfluidic channel to direct the LLS into the sample, the light sheet parameters are decoupled from light sheet steering,^33^ in contrast to in e.g., highly inclined and laminated optical sheet (HILO) and its derivatives,^34–36^ where the thickness, intensity, position, and depth of the excitation light pattern are coupled and vary depending on the incident angle of the beam. SoLLS thus enables volumetric 3D imaging throughout adherent samples with uniform sectioning. In contrast to previous works,^32,33,37^ a LLS, instead of a conventional Gaussian LS, was implemented, taking advantage of the superior characteristics of LLS and demonstrating the feasibility of structured beam illumination in soSPIM/soTILT3D geometry. We demonstrate that soLLS outperforms single-objective Gaussian LSs in terms of useful FOV and sectioning when propagating through scattering samples. Furthermore, we demonstrate that soLLS drastically reduces background fluorescence for diffraction-limited cell imaging as well as the localization precision for 2D and 3D single-molecule super-resolution cell imaging compared with conventional epi-illumination. Finally, we demonstrate that soLLS can successfully optically section through multiple cells, enabling improved two-target multicell 3D single-molecule super-resolution imaging using sequential DNA-PAINT (Exchange-PAINT)^8^—enabled and automated through the combination with microfluidics, as well as point spread function (PSF) engineering for nanoscale localization of individual molecules in 3D^38–44^ and deep learning for analysis of overlapping emitters.^45^

Taken together, the improved sectioning capability of the soLLS compared to the conventional Gaussian LS and the easy combination with microfluidics for multitarget super-resolution imaging will enable new biological discoveries by facilitating the investigation of more challenging samples.

## Results and Discussion

### Single-Objective Lattice Light Sheet (soLLS) Platform Design

To break the trade-off between LS thickness and confocal parameter of a conventional Gaussian LS, a multi-Bessel square LLS^46–49^ with suppressed side lobes was adopted to achieve extended propagation distance while retaining a thin LS for optical sectioning of multiple cells.^13,46^ To circumvent limitations of a dual-objective LLS platform, soLLS was designed with a single-objective geometry, allowing for LLS formation and fluorescence detection through the same high-NA objective lens (Figures 1a and S1). The soLLS was generated in a cost-effective and simple manner using a commercially available transmissive binary photomask^50^ for broad implementation (Figures 1b and S2). A stage-top, microfluidic chip with a metalized nanoprinted insert^33^ was then used both to function as a reflective mirror to redirect the beam for soLLS illumination and to enable precise and automated solution perfusion and exchange using microfluidics. The design of the chip insert micromirror is versatile and can be printed to either reflect the beam horizontally or angled downward at any angle to facilitate imaging of samples all the way down to the coverslip.^33,51,52^ The dimensions of the microfluidic chip can also be easily tuned to accommodate different sample sizes. Additionally, by implementing PSF engineering in the emission pathway, the soLLS setup is capable of extracting 3D information without axial scanning, providing high-precision nanoscale 3D localization of single molecules. In this work, both short- and long axial range double-helix point spread functions (DH-PSFs)^38,39,42^ were used for single-molecule localization and drift correction, respectively (Figure S3).

**Figure 1:**
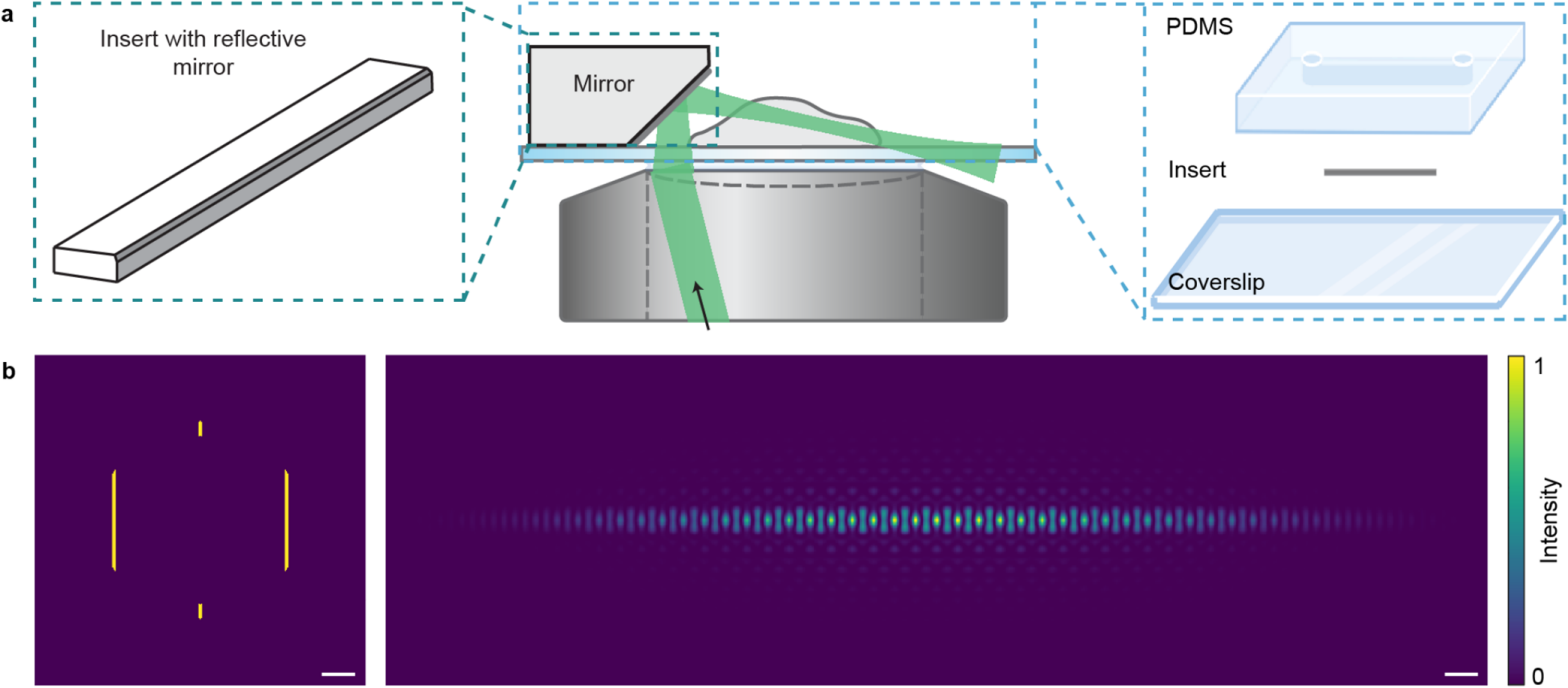
Single-objective lattice light sheet (soLLS) platform design. (a) The soLLS setup circumvents limitations of the conventional dual-objective LLS approach by implementing lattice light sheet illumination and fluorescence detection through the same high-NA objective lens. A micro-optics incorporated microfluidic chip is used for reflecting and redirecting the soLLS and for automated solution exchange. Schematics are not to scale. (b) Numerical simulations illustrating LLS generation in the setup. The input light field is shaped by a binary transmissive photomask conjugated to the back focal plane (BFP) of the objective, resulting in a LLS formed in planes conjugate to the image plane. Scale bars: left: 1 mm, right: 50 μm.

### Quantification of soLLS Characteristics

To quantify the characteristics of the soLLS, its 3D profile was imaged and compared to a single-objective Gaussian LS with comparable thickness at the focus (Figure 2a–c). The soLLS was determined to have a width of 32.9 μm (1/e^2^ radius) (Figure 2d). The thickness was determined to be 1.8 μm (1/e^2^ radius) for both the soLLS and Gaussian LS (Figure 2e), which enabled a direct comparison of their effective ranges (Figure 2f). Similar to the confocal parameter of Gaussian beams, we here define the range over which the LS thickness remains within a factor of √2 times its beam waist thickness as the effective range of the LSs. The soLLS was determined to have an effective range of 27.9 ± 4.4 μm (mean ± standard deviation, *n* = 3), thus providing a 1.5-fold improvement over the Gaussian LS that had an effective range of 19.2 ± 0.7 μm (mean ± standard deviation, *n* = 3) (Figures 2f and S4). These results demonstrate that the soLLS maintains its thin profile over a larger effective propagation range compared to a conventional Gaussian LS when initialized with a similar beam waist radius, thereby extending the imaging FOV within which the desired optical sectioning is achieved.

**Figure 2:**
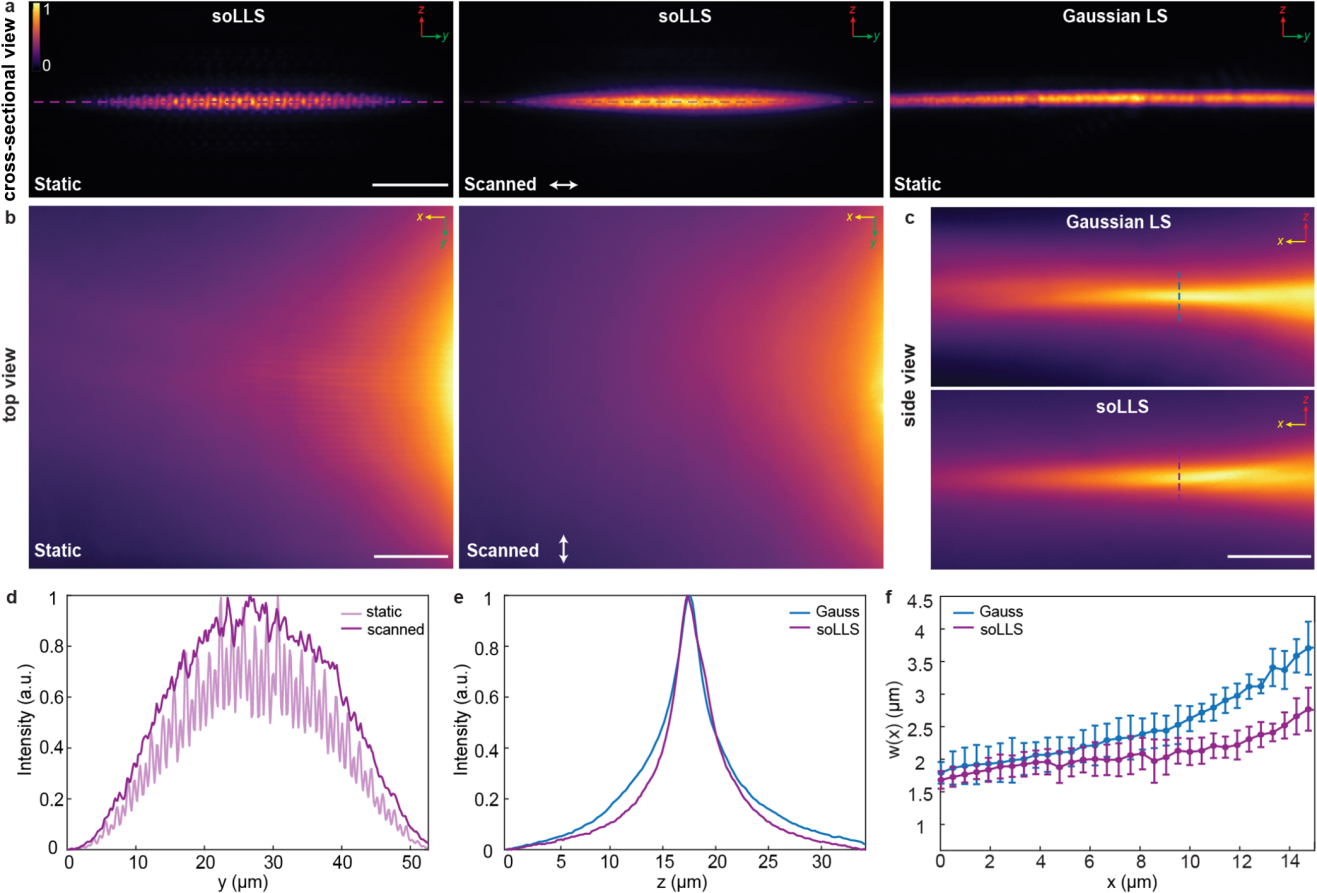
Quantification of soLLS characteristics. (a) Cross-sectional views of the soLLS profile with and without scanning along the soLLS width direction, and of a Gaussian LS with a similar thickness for comparison. Scale bar: 10 μm. (b) Top views of the soLLS profile with and without scanning along the soLLS width direction. Scale bar: 10 μm. (c) Side views of the soLLS and Gaussian LS profiles. Scale bar: 10 μm. (d) Intensity line scans of the soLLS width acquired from the top views in (b). (e) Intensity line scans of the thicknesses of the soLLS and Gaussian LS measured from the side views in (c). (f) The thicknesses (1/e^2^ radius) of the soLLS and the Gaussian LS as they propagated, measured from the side views in (c), shown as mean ± standard deviation for *n* = 3 independent measurements. The colorbar shows normalized intensity. The coordinate axes indicate the LS propagation direction (*x*), width (*y*), and thickness (*z*).

Unlike in HILO and similar designs,^34–36^ where the thickness, intensity, position, and depth of the excitation light pattern are coupled and vary depending on the incident angle of the beam, the soLLS can be repositioned in 3D over the entire imaging FOV using beam steering units.^32^ This allows the soLLS dimensions to be decoupled from the beam position, enabling consistent sectioning performance across different regions of interest. The beam steering units were calibrated by applying voltage or current signals to the galvanometric mirrors and tunable lens and measuring the resulting beam displacement. A linear response was observed between the signal input and the corresponding beam shift, allowing for intuitive and precise beam positioning during imaging. Specifically, steering axially and in the soLLS plane was implemented using galvanometric mirrors, where 0.01 V applied to the galvanometric mirrors resulted in a translation of the soLLS of 0.64 μm in the axial (*z*) direction and 0.35 μm in the direction within the soLLS plane (*y*), respectively. A tunable lens was used to shift the soLLS focus, where 10 mA applied to the tunable lens corresponded to a focus shift of 2.41 μm (Figure S5).

### SoLLS Propagates More Robustly in Scattering Environments Compared to a Gaussian LS

Self-reconstructing (or self-healing) is another defining characteristic of nondiffracting beams, referring to the ability of such beams to regenerate their initial profiles under free propagation after being disturbed by an obstacle.^53^ This property has been experimentally demonstrated for various beam types,^54–57^ and it is of particular interest in LS microscopy, as imaging of biological samples inherently suffers from scattering and aberrations due to sample heterogeneity and refractive index variations. LLS, physically described as a coherent superposition of a linear array of Bessel beams,^13^ is expected to preserve this feature to an extent depending on beam type and experimental implementation. To assess the performance of the soLLS propagation in scattering environments, we compared the soLLS and a Gaussian LS for their ability to penetrate in low-scattering fluorescent solutions (Figure 3a) and high-scattering environment generated by mixing a solution of fluorescent beads and dye in agarose gel (Figure 3b,c). We observed that the soLLS and Gaussian LS exhibit similar profiles in a low-scattering environment (Figure 3a), where the soLLS was quantified to have a 1.5-fold longer effective range (Figure 2f). In scattering environments (Figure 3b,c), the soLLS demonstrated more robust penetration. The maximum intensity projection of the Gaussian LS appears scattered and aberrated, suggesting that the observed differences are closely related to scattering, rather than being solely associated with other propagation properties. Furthermore, we quantified the ability to localize individual emitters in the scattering environment with soLLS compared with the Gaussian LS (Figure 3d–f). Our results demonstrate that, compared to a Gaussian LS, while having comparable numbers of localizations close to the beam focus, illumination with the soLLS yielded a much larger number of localizations at distances further from the beam focus, suggesting that the soLLS is more robust to aberrations caused by scattering environments (Figure 3c and Table S1). In the region furthest from beam focus (Region 5, Figure 3d), soLLS offers a 370-fold improvement in the number of detected emitters compared with the Gaussian LS (Table S1). Furthermore, the SBR values and uncertainties of the localizations obtained with soLLS illumination outperformed those obtained with Gaussian LS illumination in all regions (Figure 3e,f). Together, these results show that the soLLS behaves more robustly in complex and scattering environments, offering benefits for imaging where several tens of micrometers of light sheet penetration is required.

**Figure 3:**
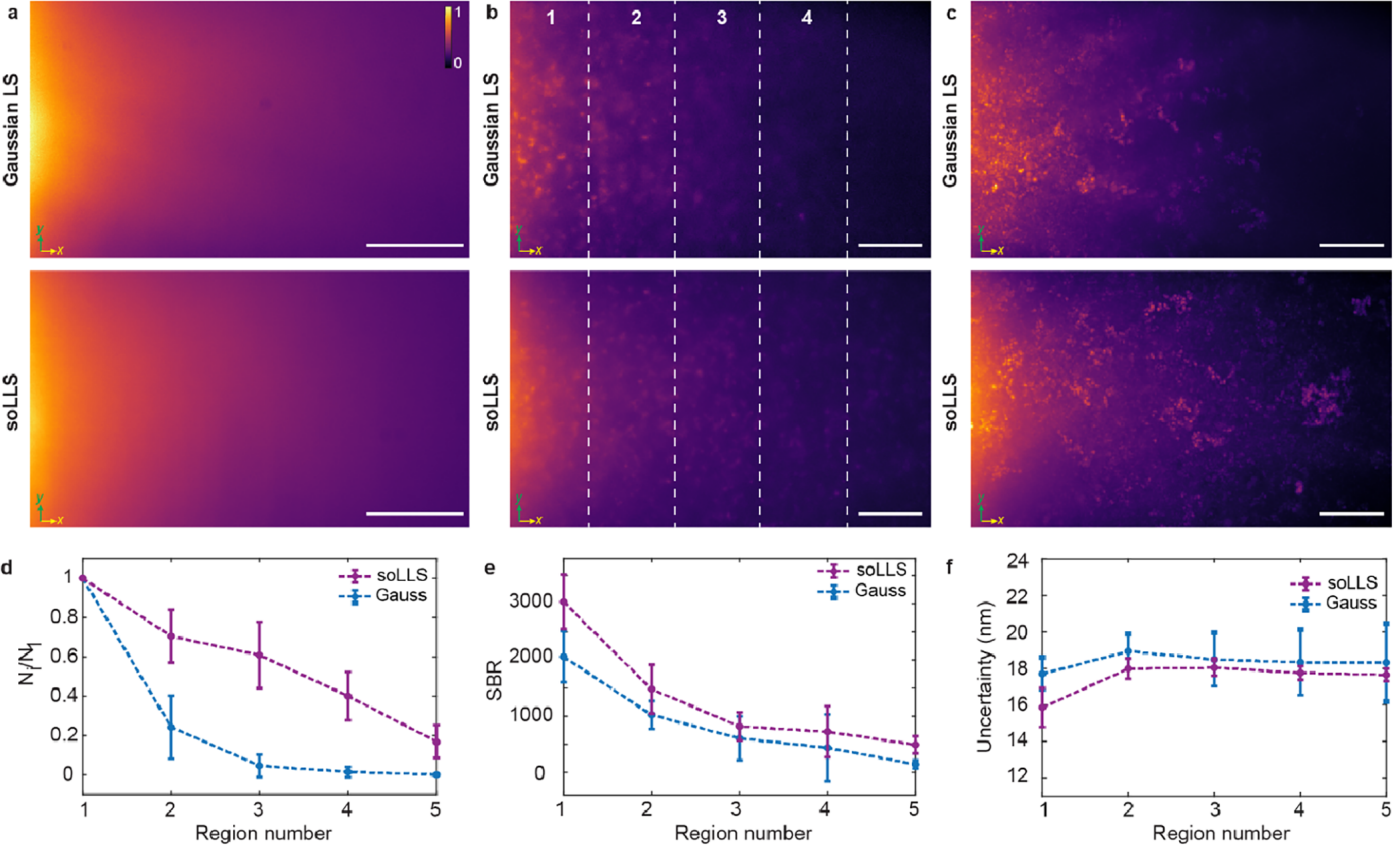
SoLLS propagates more robustly in scattering environments compared to a Gaussian LS. Representative images of the Gaussian LS (top) and soLLS (bottom) propagating through (a), a low-scattering fluorescent solution, (b) a high-scattering environment created by mixing fluorescent beads and dye in an agarose gel, and (c) maximum intensity projections of the image stacks acquired in (b). Localizations from the images were binned into 5 rectangular regions of equal area to compare statistics, with region 1 being the closest to the mirror. Scale bars: 10 μm. The colorbar shows normalized intensity. The coordinate axes indicate the LS propagation direction (*x*) and width (*y*). (d) Comparison of the number of localizations in each region, *N_i_*, normalized by the number of localizations in region 1, *N*_1_. (e) Comparison of the signal-to-background ratio (SBR) of the localizations in each region. (f) Comparison of the localization uncertainty in each region. Error bars in (d–f) represent the mean ± standard deviation of the corresponding values across *n* = 9 fields of view (FOVs). Taken together, soLLS improves both the number of detected particles, the SBR, and the localization precision compared to the Gaussian LS when imaging through scattering samples.

### SoLLS Improves the Signal-to-Background Ratio and 3D Localization Precision for Cellular Imaging

The quality of single-molecule data is often degraded when imaging throughout thick samples due to out-of-focus background fluorescence. To demonstrate that soLLS effectively optically sections thick samples, such as mammalian cells, and thereby improves the SBR for both diffraction-limited and super-resolution imaging, lamin B1 in the same U2OS cells was imaged back-to-back with soLLS and wide-field epi-illumination. This data showed that soLLS improved the SBR by up to 7.5-fold for diffraction-limited imaging (Figure 4a) and up to 3.9-fold for single-molecule imaging (Figure 4b). Technical replicates for diffraction-limited imaging (Figure S6, *n* = 3 cells) and single-molecule imaging (Figure S7, *n* = 4 cells) demonstrated reproducible performance of the soLLS across multiple samples. Next, the improvement for 3D single-molecule imaging was quantified by imaging of lamin B1 using a short-range DH-PSF with soLLS and wide-field epi-illumination. The data showed that while having more signal photons, with median values of 4320 photons/localization with soLLS illumination compared to 2654 photons/localization with epi-illumination, the median background photons were drastically reduced to 16.1 photons/pixel with soLLS illumination compared to 25.5 photons/pixel with epi-illumination (Figure 4c). This resulted in improvements in both lateral (*xy*) and axial (*z*) localization precisions, with median values of 11.9 nm laterally and 18.1 nm axially with soLLS illumination compared to 18.1 nm laterally and 27.4 nm axially with epi-illumination. Technical replicates demonstrate reproducible improvements of soLLS for 3D single-molecule imaging across multiple samples (Figure S8, *n* = 4 cells).

**Figure 4:**
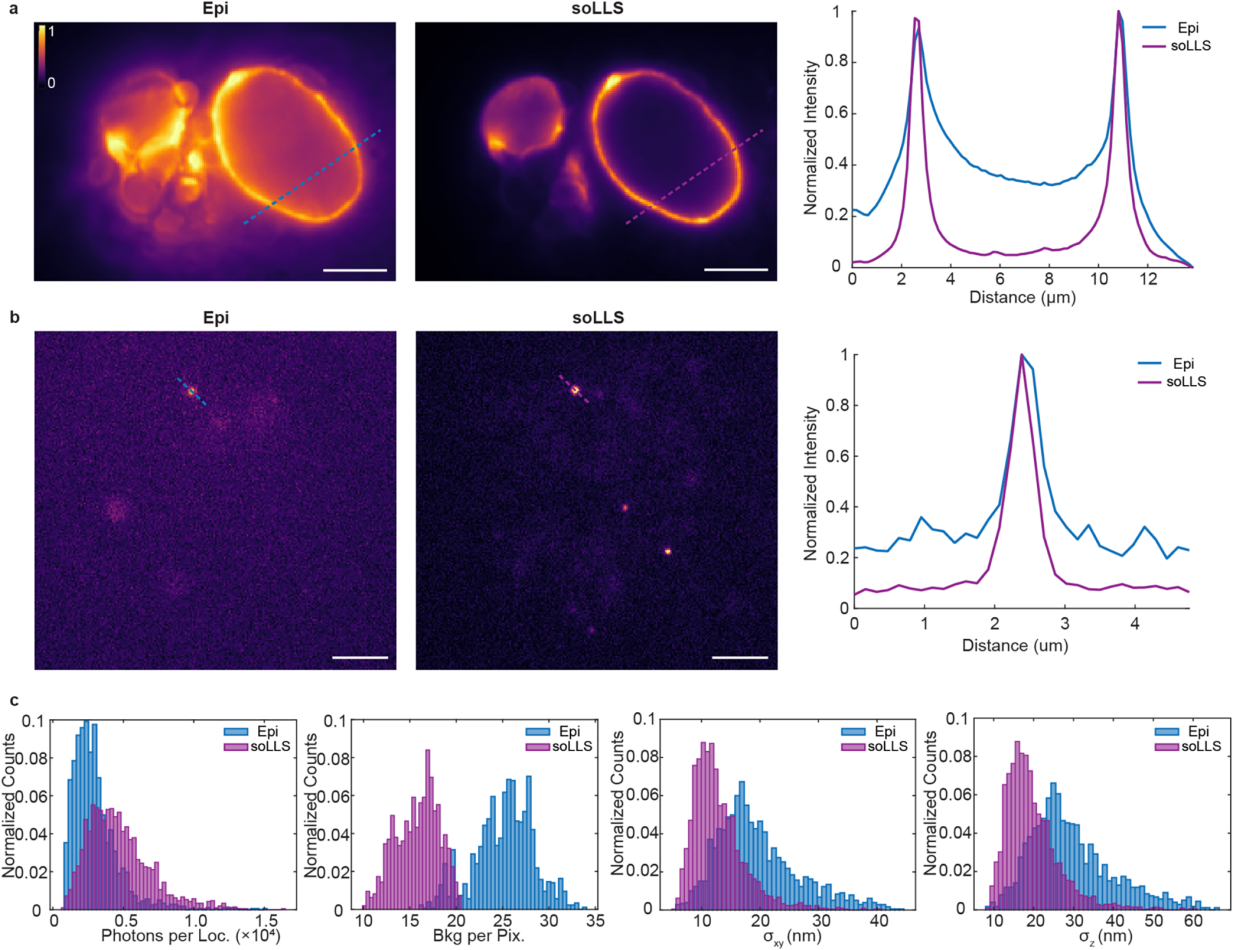
SoLLS improves the signal-to-background ratio (SBR) and 3D localization precision for cellular imaging. (a) Diffraction-limited images of lamin B1 in U2OS cells acquired with wide-field epi- (Epi) or soLLS illumination. Scale bars: 5 μm. The colorbar shows normalized intensity. The graph shows a comparison of the normalized intensity across line scans in the same cell. (b) 2D single-molecule images of DNA-PAINT labeled lamin B1 in U2OS cells acquired with epi- or soLLS illumination. Scale bars: 5 μm. The graph shows the normalized intensity across line scans of single molecules. (c) Histograms showing comparisons of signal photons per localization, background photons per pixel, lateral (*xy*) localization precision, and axial (*z*) localization precision of the localized emitters in the same cell under either epi- or soLLS illumination, demonstrating that soLLS illumination drastically reduces background photons compared to epi-illumination, which results in improved localization precision for 3D single-molecule super-resolution imaging.

Moreover, we directly compared the performance of the soLLS and the Gaussian LS when imaging through multiple cells (Figure S9), demonstrating that while both LSs provided a substantial SBR improvement over epi-illumination, soLLS outperformed the Gaussian LS by providing more uniform illumination and improved SBR for subsequent cells after passing through an upstream cell due to its superior propagation properties.

Furthermore, to demonstrate that soLLS provides useful sectioning also for live-cell imaging, we imaged mitochondria in live U2OS cells over 15 min (Figure S10). This data showed a 4.5-fold improvement in the SBR compared to epi-illumination, and the easy and rapid repositioning capabilities of soLLS ensure that sectioning is optimized throughout the time-series even during cell migration.

### SoLLS Provides Sectioning for Improved 3D Single-Molecule Super-Resolution Imaging of Multiple Targets and Multiple Cells

Light sheet sectioning through multiple cells is typically degraded due to scattering, limiting its benefits for single-molecule imaging of, for example, cell–cell junctions. Furthermore, many questions in biology and biomedicine require imaging and accurate correlation of multiple cellular structures or molecular distributions at the nanoscale, which can be hard to achieve using multicolor imaging or manual solution exchange. With soLLS, multitarget 3D single-molecule super-resolution imaging is made simple by the single-objective configuration in combination with the microfluidic chip for automated solution exchange. Having also shown that soLLS propagates more robustly through complex samples and provides excellent optical sectioning of cells, we next set out to demonstrate soLLS for 3D single-molecule super-resolution imaging of several cellular structures and throughout multiple cells. First, soLLS was demonstrated for two-target Exchange-PAINT imaging of lamina-associated protein 2 (LAP2) and mitochondria (TOMM20) using the microfluidic chip for automated solution exchange (Figure 5a–c). The resulting Fourier ring correlation (FRC) resolutions in the *xy*/*xz*/*yz* planes were found to be 19.2/23.4/23.4 nm for LAP2 and 24.2/30.8/28.5 nm for TOMM20 (Figures 5d and S11), demonstrating that soLLS achieves multitarget imaging with high spatial resolution in 3D (Figure 5e).

**Figure 5:**
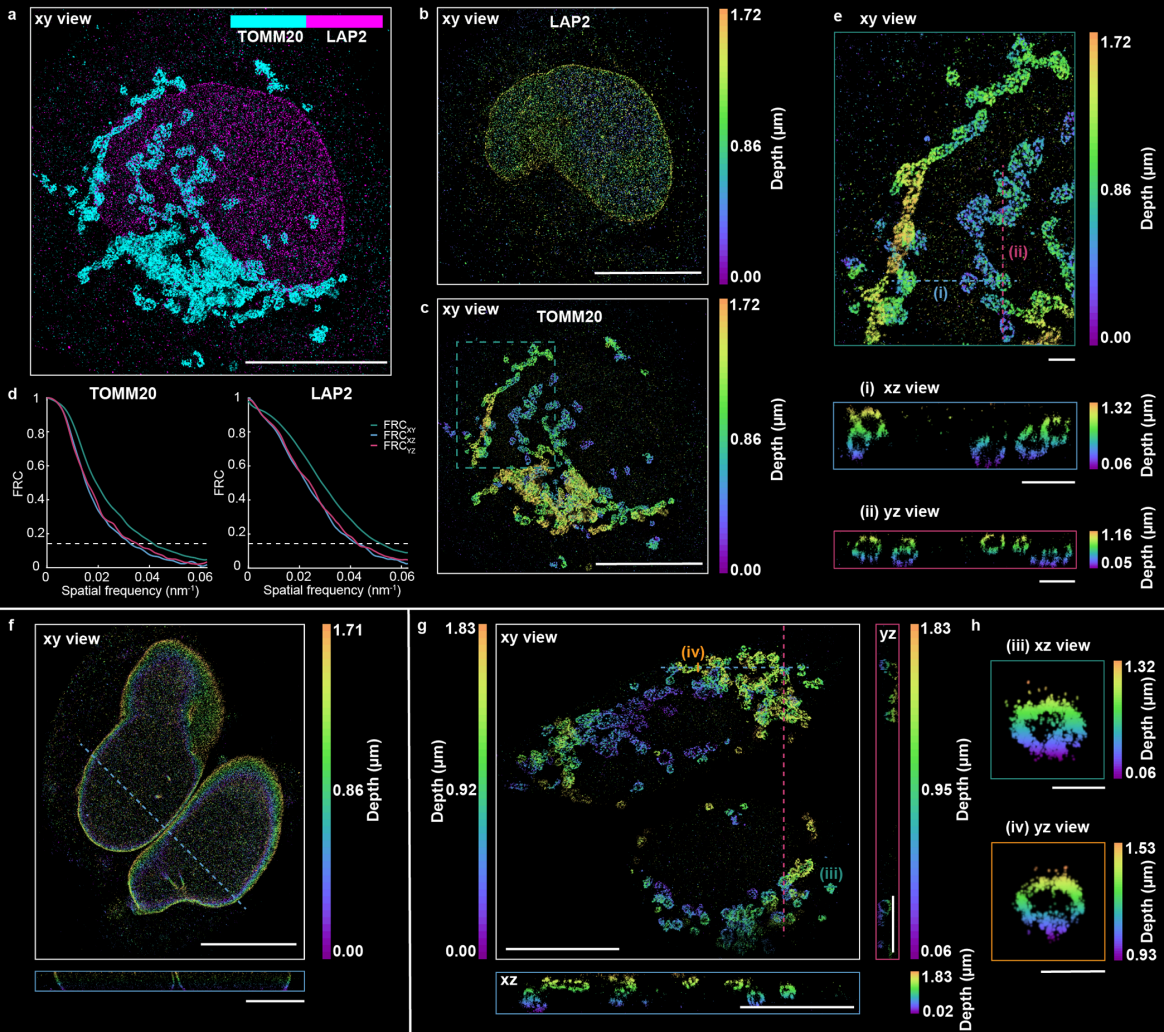
SoLLS provides sectioning for improved 3D single-molecule super-resolution imaging of multiple targets and multiple cells. (a) Two-target 3D super-resolution reconstruction of LAP2 and mitochondria (TOMM20) in the same U2OS cell, colored by target. Scale bar: 10 μm. Separate renderings of (b) LAP2 and (c) TOMM20, colored by depth. Scale bars: 10 μm. (d) The resulting FRC resolutions in the xy/xz/yz planes for LAP2 and TOMM20. (e) Zoomed-in views of the mitochondria in the dashed box in (c). The xz and yz views are shown for a 200 nm thick y-slice in the xz plane and a 200 nm thick x-slice in the yz plane at the dashed lines (i) and (ii) in the xy view. Scale bars: 1 μm. (f, g), SoLLS sectioning and 3D super-resolution imaging in two adjacent cells are shown for (f) lamin A/C and (g) mitochondria (TOMM20). Scale bars: 10 μm in the xy views and 5 μm in the cross-sectional views. (h) Zoomed-in views of mitochondria cross sections acquired along the solid lines (iii) and (iv) in (g), 300 and 200 nm thick, respectively. Scale bars: 500 nm.

Next, by leveraging the enhanced propagation performance of the soLLS, multiple cells were successfully sectioned and imaged across the same FOV, which is typically challenging for conventional Gaussian LSs of similar thickness. Simultaneous 3D single-molecule super-resolution imaging of two cells, labeled for either lamin A/C (Figure 5f) or mitochondria (Figure 5g,h), was achieved with resulting FRC resolutions in the xy/xz/yz planes of 33.1/45.1/45.0 nm for lamin A/C and 25.1/33.6/29.5 nm for the mitochondria (Figure S11), demonstrating that the high spatial resolution is retained even when illuminating through multiple cells.

## Conclusions

In this work, we presented soLLS, an imaging platform that implements a reflected single-objective lattice light sheet generated by a simple printed photomask in combination with a micro-optic incorporated into a microfluidic chip for reflection of the light sheet and for easy and automated solution exchange. The single-objective geometry enables LLS illumination and fluorescence detection through the same high-NA objective, eliminating limitations of the conventional dual-objective LLS design. We provided quantitative experimental characterization of the soLLS profile in 3D and compared its propagation properties to a Gaussian LS with the same thickness. We demonstrated that soLLS provides optical sectioning over a 1.5-fold longer effective range compared to a conventional Gaussian LS, due to reduced divergence of its main lobe during propagation. Further improvement in the propagation distance is expected to be achieved by increasing the optical density of the photomask to eliminate any Gaussian features arising from unmodulated light. The generation of the optical lattice is dependent on the precise alignment of the laser beam throughout the optical path, including through the photomask. Although the implementation of a photomask for pattern generation is cost-effective and convenient, it results in noticeable power loss, which can be mitigated by using an axicon to redistribute the intensity that illuminates the photomask slits,^50^ or replacing the photomask with phase-modulation masks^58^ or devices.^13,59^

Moreover, we demonstrated that the soLLS propagates more robustly in highly scattering environments, yielding up to 370-fold more localizations further away from the beam focus. Furthermore, we demonstrated an up to 7.5-fold SBR improvement for diffraction-limited imaging and a 3.9-fold SBR improvement for single-molecule imaging. The excellent sectioning by soLLS improved the 3D single-molecule localization precision from 20 to 13 nm laterally and from 30 to 20 nm axially by reducing out-of-focus background photons. Next, we demonstrate soLLS for 3D single-molecule super-resolution imaging of two targets through Exchange-PAINT with automated solution exchange, which is typically challenging with conventional LLS designs. Finally, we showcase simultaneous 3D single-molecule super-resolution imaging of multiple cells across the FOV, which can typically not be sectioned well with regular Gaussian LSs.

Previous work demonstrated single-objective Airy light sheet imaging (SoALSI),^36^ where a rotating mirror was used to incline an Airy beam relative to the sample plane and the self-accelerating property of Airy beams contributed to the inclination angle. This approach resulted in a 5-fold larger imaging FOV compared to when using a Gaussian LS and an up to 1.3-fold inclination-dependent SBR improvement over epi-illumination. Compared to this design, our platform is fundamentally different in its optical geometry by implementing a reflective micromirror inside a microfluidic chip to redirect the beam, resulting in a much larger and inclination-independent SBR improvement in 3D and over the whole FOV.

The soLLS imaging platform can be applied to address a large variety of biological and biomedical questions. The extended imaging FOV enabled by the soLLS platform facilitates imaging of, e.g., intercellular proteins in multiple cells or mitotic cells, and of molecular 3D distributions in cell–cell junctions.^60–63^ The soLLS platform is live-cell compatible and can be used with conventional stage-top incubators, enabling fast, precise, and gentle 3D single-particle tracking in live cells. Here, we present a demonstration of live-cell mitochondrial imaging using the soLLS platform (Figure S10), which shows improved SBR and rapid repositioning of the soLLS. These capabilities facilitate fast live-cell imaging and open up a wide range of future live-cell applications.^64–67^ The microfluidic chip simplifies the process of introducing buffers and solutions to the sample, which facilitates multitarget Exchange-PAINT imaging extended beyond the two-target imaging demonstration presented in this work.^8,33^ Additionally, it enables well-controlled and reversible introduction of drugs or other stimuli for live-cell studies.^68–72^ The fabrication of the reflective insert involves two-photon polymerization and metal coating through deposition. Simplifying this fabrication process in the future will enable an even broader implementation. While the current soLLS setup is optimized for imaging multiple cells in terms of soLLS characteristics, sample chamber sizes, and beam shaping optics, the design can be adapted for larger-scale samples such as organoids by the implementation of a silicone oil immersion objective,^73^ adaptive optics in both the excitation and emission paths,^74,75^ and straightforward changes in the dimensions of the reflective insert and the microfluidic chip. Taken together, soLLS offers a powerful, flexible, and cost-effective approach for improved 3D single-molecule super-resolution imaging of mammalian cells. SoLLS can be utilized for various single-molecule imaging applications for improved nanoscale investigations of cellular architectures and molecular dynamics.

## Experimental Methods

### Single-Objective Lattice Light Sheet (soLLS) Imaging Platform

The soLLS platform was built around an inverted microscope (IX83, Olympus) with a high-NA oil immersion objective (UPLXAPO100X, 100×, NA 1.45, Olympus) (Figure S1 and Table S2). The excitation laser (560 nm, software-controlled power range: 150–1000 mW, MPB Communications) was spectrally filtered (FF01–554/23–25, Semrock) and circularly polarized using a polarizer (LPVISC050MP2, Thorlabs) and a quarter-wave plate (Z-10-A.250-B-556, Tower Optical). Neutral density filters (NE01B-NE50B, Thorlabs) were mounted in a filter wheel (FW2A, Thorlabs) and used to adjust the illumination intensity. The laser was collimated and expanded to a desired spot size by using multiple lens telescopes. The LLS was generated with a custom-designed photomask (optical density: 5.2 at 560 nm, HTA photomask) conjugated to the back focal plane (BFP) of the objective. The photomask had multiple patterns for flexible tuning of the LLS dimensions and accommodation to different laser spot sizes in the optical path. The characteristics of the LLS are determined by the dimensions of the slit pattern. In this study, we employed a pattern that has a distance of 2.4 mm between the outer slits and a slit width of 0.08 mm (Figure S2). Two galvanometric mirrors (GVS011, Thorlabs) and a tunable lens (EL-3-10-VIS-26D-FPC, Optotune) were aligned and conjugated to the BFP of the objective lens to function as beam steering units. In addition to positioning the soLLS, the galvanometric mirror used for translating the soLLS in the soLLS plane was scanned with a sine signal at 20 Hz with a 0.1 V amplitude to generate a uniform illumination profile. Additionally, a dithering mirror (SP30Y-AG, Thorlabs) conjugated to the image plane was used to dither the soLLS with a sine signal at 100 Hz with 0.1 V amplitude to remove any residual shadowing artifacts.^76^ After propagating vertically after the objective, the soLLS was reflected and redirected by a microfluidic chip with an insert that functioned as a reflective mirror. The microfluidic chip was fabricated as detailed in ref 33. In brief, an insert with custom-designed dimensions and an angled side wall was printed using two-photon polymerization (Nanoscribe Photonic Professional GT2, Nanoscribe GmbH & Co.KG), and the side wall was metalized (E-beam Evaporator, Sharon) by e-beam deposition of 200 nm of silica (EVMSIO21–5D, Kurt J. Lesker), 350 nm of aluminum (EVMAL50EXEB, Kurt J. Lesker), and followed by 5 nm of silica (EVMSIO21–5D, Kurt J. Lesker) to function as a reflective mirror. The insert was assembled with a PDMS base and a glass coverslip. To ensure that the insert is properly aligned, the height of all inserts was made to match the depth of the microfluidic channel, and the inserts were positioned flush with the boundary of the microfluidic channel, with full contact and correct orientation. The chip was then plasma-bonded with oxygen to serve as the sample chamber. The dimensions of the chip can be easily modified to facilitate in-chip cell culture of samples with varying sizes and to enable different illumination geometries. The chip can be cleaned and reused as needed until degradation of the insert occurs due to exposure to cleaning chemicals.

For comparison between the soLLS and the conventional Gaussian LS, a single-objective Gaussian LS was generated with a cylindrical lens and aligned to have a similar thickness at focus as the soLLS. The Gaussian LS shares the beam steering units with the soLLS. The linear response of the galvanometric mirrors was calibrated separately,^33^ and 2x difference was seen between the soLLS and the Gaussian beam due to the additional beam-shrinking 4f telescope in the soLLS path. The angle of the reflective insert used to tilt both the soLLS and Gaussian LS was optimized to be 39°, enabling optical sectioning of U2OS cells with both LSs (Figure S9), while avoiding reflections and aberrations from the coverslip. Additionally, a wide-field epi-illumination path was integrated into the imaging platform for comparisons between different illumination modalities and overall flexibility and versatility of the setup. Switching between different illumination modalities was enabled using several flip mirrors, such that the laser beam is sent to only one path at a time (Figure S1). The overall transmission efficiency from the laser output to the sample plane is approximately 6.4% for the soLLS and 32.5% for the Gaussian LS, with the main source of power loss in the soLLS path being the photomask. Irises were carefully positioned along the illumination paths to ensure proper alignment, and the positions of both LSs were checked prior to each experiment to confirm the alignment.

The fluorescence emission from the sample was collected with the same objective lens and focused with the tube lens inside the microscope. The collected emission light then passed through the first lens of a 4f-system before being split by a dichroic mirror (T660lpxr, Chroma) into two channels. Each of the channels had a second 4f lens that focused the light onto an EMCCD camera (iXon Ultra 897, Andor). The 4f systems provide access to the Fourier plane in each channel to enable phase modulations for PSF engineering, where commercially available DH phase masks (short-range: DH1–580, long-range: DH12R-670, both Double Helix Optics, Inc.)^42^ were aligned. In this work, fiducial beads emitting in a separate channel from the single molecules were used, enabling independent modulation of their emission with a long-range DH phase mask to accommodate longer acquisition times or correction of more severe drift.

### Sample Preparation

For characterization of the soLLS, a coverslip spin-coated with donkey antirabbit secondary antibodies conjugated with dye CF568 (20098–1, Biotium) at a dilution of 1:10 in 1% (w/v) poly­(vinyl alcohol) (PVA) prepared in nanopure water was used to visualize the cross-sectional profile of the soLLS and the Gaussian LS. The propagation profiles of the LSs were visualized using a microfluidic chip with a reflective mirror at a 45° angle filled with 0.2 mg/mL CF568 in nanopure water.

For quantification of the soLLS propagation in scattering environments, agarose powder (Sigma-Aldrich A9539) was dissolved in nanopore water to create a solution containing 1% (w/v) agarose gel. 10 μL of this gel was then mixed with 10 μL of fluorescence beads (F8800, Invitrogen) at a 1:10 dilution in nanopure water at 95 °C and 10 μL of 0.04 mg/mL CF568 in nanopure water, which served as additional fluorescent background. The mixed solution was then dispensed into a microfluidic chip with a reflective mirror at a 45° angle right before imaging.

For cell experiments, human osteosarcoma cells (U-2 OS HTB-96, ATCC) were cultured and fixed inside microfluidic chips with a reflective mirror at a 39° angle. The microfluidic channels were cleaned with 70% ethanol (BP82031GAL, Fisher Scientific) for 10 min, followed by washing with phosphate-buffered saline (PBS) (SH3025601, Fisher Scientific) six times and then coated with a 0.002% (w/v) solution of fibronectin (F0895, Sigma-Aldrich) in PBS for 2 h. U2OS cells were then seeded into the chips and incubated at 37 °C and 5% carbon dioxide for 8 h before fixation. For fixation, cells were washed three times in PBS, fixed for 20 min in chilled 4% (w/v) formaldehyde (Electron Microscopy Science) dissolved in PBS, and then incubated for 10 min with 10 mM ammonium chloride (Sigma-Aldrich) in PBS.

For benchmarking the background reduction with soLLS, U2OS cells were labeled for lamin B1. After fixation, cells were permeabilized with three washes of 0.2% (v/v) Triton X-100 in PBS (5 min each) and subsequently blocked with 3% (w/v) bovine serum albumin (BSA) in PBS for 1 h. For both diffraction-limited and DNA-PAINT imaging of lamin B1, cells were incubated with rabbit antilamin B1 primary antibodies (ab16048, Abcam) at a 1:1000 dilution in 1% (w/v) BSA in PBS for 2 h, followed by three washes with 0.1% (v/v) Triton X-100 in PBS (3 min each). For diffraction-limited imaging, cells were incubated with donkey antirabbit secondary antibodies conjugated with CF568 (20098–1, Biotium) at a 1:100 dilution in 1% (w/v) BSA in PBS for 1 h, then washed five times with 0.1% (v/v) Triton X-100 in PBS. For DNA-PAINT imaging, cells were incubated with donkey antirabbit secondary antibodies conjugated to oligonucleotides (Massive Photonics, order number AB2401012) at a 1:100 dilution in antibody incubation buffer (Massive Photonics) for 1 h, followed by three washes with 1× washing buffer (Massive Photonics) in nanopure water. Complementary oligonucleotide-Cy3B dye conjugates (Massive Photonics, order number AB2401012) were diluted in imaging buffer (500 mM NaCl in PBS, pH 8) to a final concentration of 0.1 and 0.01 nM for 2D and 3D single-molecule imaging, respectively. For 3D imaging, the solutions were introduced into the chip at 50 mbar, corresponding to a flow rate of 7.4 μL/min, using a pressure-based flow control pump (LU-FEZ-0345, Fluigent, Inc.).

For 3D DNA-PAINT super-resolution imaging of TOMM20, LAP2, and lamin A/C, the cells were permeabilized in 0.1% (w/v) saponin (SAE0073, Sigma-Aldrich) in PBS for 10 min, and blocked with 10% (v/v) donkey serum and 0.05 mg/mL salmon sperm DNA in 0.1% (w/v) saponin in PBS for 1.5 h. For two-target TOMM20 and LAP2 imaging, the cells were labeled with rabbit anti-TOMM20 primary antibodies (ab186735, Abcam) and goat antithymopoietin (AF843, R&D Systems) primary antibodies at a dilution of 1:200 and 1:50, respectively, in 10% (v/v) donkey serum and 0.05 mg/mL salmon sperm ssDNA in 0.1% (w/v) saponin in PBS for 1 h. Cells were then washed three times with PBS (5 min each). After that, the cells were labeled with donkey antirabbit and donkey antigoat oligonucleotide-conjugated secondary antibodies (Massive Photonics, order number AB2401012) at a dilution of 1:100 in antibody incubation buffer (Massive Photonics) for 1 h. For lamin A/C imaging, cells were labeled with mouse antilamin A/C primary antibodies (sc-376248, Santa Cruz Biotechnology) at a dilution of 1:100 in 10% (v/v) donkey serum and 0.05 mg/mL salmon sperm ssDNA in 0.1% (w/v) saponin in PBS for 1 h. After three washes with PBS, the cells were labeled with donkey antimouse oligonucleotide-conjugated secondary antibodies (Massive Photonics, order number AB2401012) at a dilution of 1:100 in antibody incubation buffer (Massive Photonics) for 1 h.

After labeling, the cells were washed three times with PBS and three times with 1× washing buffer (Massive Photonics) in nanopure water. Fiducial beads (F8807, Invitrogen) for drift correction were added to the chip at a dilution of 1:100,000 in PBS and incubated for 20 min before imaging. After fiducial beads were allowed to settle and attach, the cells were washed three times with 1× washing buffer.

During imaging, complementary oligonucleotide-Cy3B dye conjugates (Massive Photonics, order number AB2401012) diluted in imaging buffer (500 mM NaCl in PBS, pH 8) were introduced into the chip at 100 mbar (corresponding to a flow rate of 14.9 μL/min) using the pump. For two-target imaging, the corresponding solutions were introduced at concentrations of 0.04 nM for TOMM20 and 0.08 nM for LAP2. For single-target imaging of TOMM20 or lamin A/C, the corresponding solutions were introduced at a concentration of 0.04 nM.

For live-cell imaging, U2OS cells were seeded inside a microfluidic chip with incubation at 37 °C and 5% carbon dioxide for 6 h before labeling with 100 nM MitoTracker (M7510, Invitrogen) diluted in cell media for 30 min. The sample was then washed with cell media, placed inside a stage-top incubator (Okolab, H301PI736ZR1/2S), and maintained at 37 °C and 5% carbon dioxide at 90% humidity.

### Acquisition Settings

For all imaging, an EMCCD camera (iXon Ultra 897, Andor) was used for data acquisition. An EM gain set at 200 was used, corresponding to a calibrated EM gain of 182 for the EMCCD camera. The conversion gain of the EMCCD was experimentally determined to be 4.41 photoelectrons per A/D count. The EMCCD was operated at a shift speed of 3.3 μm/s with a standard vertical clock voltage amplitude. The readout rate was set to 17 MHz at 16-bit resolution using a preamplifier gain of 3. The calibrated pixel size of the camera was 159 nm/pixel vertically and 157 nm/pixel horizontally.

To enable a direct comparison between different illumination strategies, the power settings in the laser control software and the neutral density (ND) filters in the imaging setup were adjusted to experimentally match the signal photon counts, which were monitored in the camera software during acquisition and compared during postprocessing.

For characterization of the soLLS, image stacks of 200 frames of the cross-sectional views and 100 frames of the top and side views were acquired with a 560 nm laser at 0.2 W/cm^2^ and an exposure time of 100 ms. For the Gaussian LS, image stacks of 50 frames of the cross-sectional view and 100 frames of the side view were acquired with a 560 nm laser at 0.1 W/cm^2^ and an exposure time of 100 ms.

For propagation of the LSs in scattering environments, image stacks consisting of 500 frames were sequentially acquired from nine different FOVs for each condition (soLLS and Gaussian LS), using an exposure time of 100 ms. The 560 nm excitation laser was used at 7.2 W/cm^2^ for soLLS illumination and 13.6 W/cm^2^ for Gaussian LS illumination.

For diffraction-limited imaging of lamin B1, image stacks of 100 frames at an exposure time of 100 ms were acquired with a 560 nm laser at ∼1 W/cm^2^ for both soLLS and epi-illumination. For 2D single-molecule imaging of lamin B1, image stacks of 5000 frames at an exposure time of 200 ms were acquired with the 560 nm laser at ∼120 and ∼460 W/cm^2^ for soLLS and epi-illumination, respectively. For 3D single-molecule imaging of lamin B1, image stacks of 1000 frames at an exposure time of 200 ms were acquired with the 560 nm laser at ∼160 and ∼570 W/cm^2^ for soLLS and epi-illumination, respectively.

For 3D super-resolution imaging of TOMM20, LAP2, and lamin A/C, a 560 nm laser was used at 415 W/cm^2^ for soLLS illumination. For two-target imaging, image stacks of 60,000 frames were acquired for TOMM20 and 55,000 frames for LAP2 at an exposure time of 200 ms. For single-target imaging of TOMM20, image stacks of 150,000 frames were acquired at an exposure time of 200 ms. For single-target imaging of lamin A/C, image stacks of 50,000 frames were acquired at an exposure time of 200 ms.

For live-cell imaging, image stacks of 50 frames were acquired with 0.1 W/cm^2^ 560 nm illumination and an exposure time of 100 ms every 30 s over a 15 min time period.

### Data Analysis

For the characterization of the LS profiles, diffraction-limited images were analyzed using ImageJ to extract intensity distributions and fitting results of the LS dimensions. Even though soLLS sections well also close to the reflective mirror, due to the single-objective geometry and the experimental procedure of visualizing the beam in fluorescent solution, the region near the reflective mirror (before the beam focus) shows high fluorescence in characterization images, which compromises measurements of the beam profile in that region. For accurate measurements of the beam profile, quantification was therefore performed after beam focus.

For comparing the soLLS and Gaussian LS in the scattering environments, image stacks of *n* = 9 different FOVs were acquired under soLLS and Gaussian LS illumination and were cropped to the same size for postprocessing. Image stacks were analyzed in ThunderSTORM,^77^ an open-source ImageJ plugin, which localizes the single emitters by local maxima detection and weighted least-squares fitting and calculates the signal photon counts and background counts per pixel of each emitter. The localization precision was calculated from these fitted parameters using the EMCCD-specific formula.^78^ Specifically, data were processed with wavelet filtering (B-Spline order of 3 and scale of 2.0) and a weighted least-squares fitting. PSFs were detected using a maximum fitting method with a peak intensity threshold coefficient of 2. After filtering out low-confidence localizations (*vide infra*), the remaining localizations were binned into 5 rectangular regions of equal area for statistics comparison. The regions were labeled 1 to 5, with region 1 being closest to and region 5 being furthest away from the beam focus.

For benchmarking the improvement in single-molecule imaging with soLLS, intensity line plots of the emitters were acquired from 2D single-molecule images of the same cell illuminated with soLLS or epi-illumination. The mean background reduction ratio between soLLS and epi-illumination was determined from intensity line scans over a 1.6 μm range where the background was measured, and the improvement in SBR was determined by averaging the mean ratio for *n* = 3 emitters for each cell. 3D single-molecule data for lamin B1 were analyzed with a modified version of Easy-DHPSF,^79^ which localizes the single-molecule data in 3D and provides the signal and background photon counts by a 2D double-Gaussian fit. The 3D localization precision of each localization was calculated in the software using an analytical expression incorporating fitted parameters.^80^

For 3D super-resolution imaging of TOMM20, LAP2, and lamin A/C, single-molecule data acquired with the short-range DH-PSF were localized using DECODE, an open-source deep learning-based software capable of localizing dense and overlapping emitters.^33,45^ The 3D position, signal, background, and localization precision were obtained directly from DECODE’s neural network output. The DECODE model was trained using a z-scan of fluorescent beads imaged with the short-range DH-PSF and sparse 3D single-molecule data acquired from the same experimental setup, with setup-specific parameters: a dark level of 500 A/D counts, a conversion gain of 4.4 photoelectrons per A/D count, and an EM gain of 181.8. Training stopped after convergence to a Jaccard index of 0.68 after 1000 epochs. The trained model was suitable for detecting signal photons in the range of 0–29,700 photons per localization and background levels of 0 to 240 photons per pixel. The trained model was then used for analyzing the various 3D single-molecule data sets throughout this work. The localizations were then drift-corrected using a custom MATLAB script with respect to the localizations of the fiducial beads within the FOV, which were acquired with the long-range DH-PSF and localized with a modified version of Easy-DHPSF.

### Filtering, Rendering, and Resolution Analysis

Localizations were filtered to remove low-confidence localizations. For the soLLS versus Gaussian LS comparison in scattering environments, localizations with uncertainty larger than 100 nm or photon counts exceeding 1,000,000 photons per localization were discarded to remove outliers.

For 3D super-resolution imaging of TOMM20, LAP2, and lamin A/C, the localized data were filtered and rendered using the Vutara SRX (Bruker) software. For the two-target TOMM20 and LAP2 reconstruction, all TOMM20 localizations with localization precision greater than 42 nm in *xy*, greater than 50 nm in *z*, or having a signal photon count lower than 3000 were removed. LAP2 localizations with a localization precision greater than 90 nm in *xy*, greater than 92 nm in *z*, or having a signal photon count lower than 500 were removed. For single-target multicell imaging of TOMM20, localizations with localization precision greater than 20 nm in *xy* or greater than 40 nm in *z* were removed. For single-target multicell imaging of lamin A/C, localizations with localization precision greater than 40 nm in *xy* or greater than 90 nm in *z* were removed. After filtering, all *z* localizations of the remaining localizations were scaled by a factor of 0.75 to account for index mismatch between the glass coverslip and the sample.^33,67,81–83^

The 3D super-resolution reconstructions were visualized by point splat rendering using a 25 nm particle size in Vutara SRX.

For resolution analysis, the FRC values were calculated in Vutara SRX using a super-resolution pixel size of 8 nm.

## Supplementary Material



## Data Availability

Source data for all graphs presented in this study are provided and will be made publicly available on GitHub upon completion of the peer review and publication process: [https://github.com/Gustavsson-Lab/soLLS-SourceData].

## Notes

Schematic of the optical setup (Figure S1); photomask design (Figure S2); double-helix PSFs (Figure S3); additional characterization of beam thickness (Figure S4); calibration of the steering units (Figure S5); technical replicates for Figure 3 (Figure S6–S8); comparison between soLLS and Gaussian LS (Figure S9); live-cell demonstration (Figure S10); FRC analysis for the 3D reconstructions (Figure S11); absolute number of localizations for Figure 2 (Table S1); and components list of the optical setup (Table S2) (PDF)

## References

[1] Lichtman J. W., Conchello J.-A. (2005). Fluorescence
Microscopy. Nat.
Methods.

[2] Hickey S. M., Ung B., Bader C., Brooks R., Lazniewska J., Johnson I. R. D., Sorvina A., Logan J., Martini C., Moore C. R., Karageorgos L., Sweetman M. J., Brooks D. A. (2022). Fluorescence
Microscopy—An Outline of Hardware, Biological Handling, and
Fluorophore Considerations. Cells.

[3] Sharonov A., Hochstrasser R. M. (2006). Wide-Field
Subdiffraction Imaging by Accumulated Binding
of Diffusing Probes. Proc. Natl. Acad. Sci.
U. S. A..

[4] Betzig E., Patterson G. H., Sougrat R., Lindwasser O. W., Olenych S., Bonifacino J. S., Davidson M. W., Lippincott-Schwartz J., Hess H. F. (2006). Imaging Intracellular Fluorescent Proteins at Nanometer
Resolution. Science.

[5] Rust M. J., Bates M., Zhuang X. (2006). Sub-Diffraction-Limit
Imaging by
Stochastic Optical Reconstruction Microscopy (STORM). Nat. Methods.

[6] Hess S. T., Girirajan T. P. K., Mason M. D. (2006). Ultra-High Resolution Imaging by
Fluorescence Photoactivation Localization Microscopy. Biophys. J..

[7] Jungmann R., Steinhauer C., Scheible M., Kuzyk A., Tinnefeld P., Simmel F. C. (2010). Single-Molecule Kinetics and Super-Resolution Microscopy
by Fluorescence Imaging of Transient Binding on DNA Origami. Nano Lett..

[8] Jungmann R., Avendaño M. S., Woehrstein J. B., Dai M., Shih W. M., Yin P. (2014). Multiplexed
3D Cellular Super-Resolution Imaging with DNA-PAINT and
Exchange-PAINT. Nat. Methods.

[9] Power R. M., Huisken J. (2017). A Guide to Light-Sheet
Fluorescence Microscopy for
Multiscale Imaging. Nat. Methods.

[10] Gustavsson A.-K., Petrov P. N., Moerner W. E. (2018). Light Sheet
Approaches for Improved
Precision in 3D Localization-Based Super-Resolution Imaging in Mammalian
Cells [Invited]. Opt. Express.

[11] Gagliano G., Nelson T., Saliba N., Vargas-Hernández S., Gustavsson A.-K. (2021). Light Sheet Illumination for 3D Single-Molecule Super-Resolution
Imaging of Neuronal Synapses. Front. Synaptic
Neurosci..

[12] Cheng S., Nakatani Y., Gagliano G., Saliba N., Gustavsson A.-K. (2024). Light Sheet
Illumination in Single-Molecule Localization Microscopy for Imaging
of Cellular Architectures and Molecular Dynamics. npj Imaging.

[13] Chen B.-C., Legant W. R., Wang K., Shao L., Milkie D. E., Davidson M. W., Janetopoulos C., Wu X. S., Hammer J. A., Liu Z., English B. P., Mimori-Kiyosue Y., Romero D. P., Ritter A. T., Lippincott-Schwartz J., Fritz-Laylin L., Mullins R. D., Mitchell D. M., Bembenek J. N., Reymann A.-C., Böhme R., Grill S. W., Wang J. T., Seydoux G., Tulu U. S., Kiehart D. P., Betzig E. (2014). Lattice Light-Sheet
Microscopy: Imaging
Molecules to Embryos at High Spatiotemporal Resolution. Science.

[14] Liu G., Ruan X., Milkie D. E., Görlitz F., Mueller M., Hercule W., Killilea A., Betzig E., Upadhyayula S. (2023). Characterization, Comparison, and
Optimization of Lattice
Light Sheets. Sci. Adv..

[15] Planchon T. A., Gao L., Milkie D. E., Davidson M. W., Galbraith J. A., Galbraith C. G., Betzig E. (2011). Rapid Three-Dimensional Isotropic
Imaging of Living Cells Using Bessel Beam Plane Illumination. Nat. Methods.

[16] Heilemann M., van de Linde S., Schüttpelz M., Kasper R., Seefeldt B., Mukherjee A., Tinnefeld P., Sauer M. (2008). Subdiffraction-Resolution
Fluorescence Imaging with Conventional Fluorescent Probes. Angew. Chem., Int. Ed..

[17] Legant W. R., Shao L., Grimm J. B., Brown T. A., Milkie D. E., Avants B. B., Lavis L. D., Betzig E. (2016). High-Density Three-Dimensional
Localization Microscopy across Large Volumes. Nat. Methods.

[18] Wäldchen F., Schlegel J., Götz R., Luciano M., Schnermann M., Doose S., Sauer M. (2020). Whole-Cell Imaging of Plasma Membrane
Receptors by 3D Lattice Light-Sheet dSTORM. Nat. Commun..

[19] Liu T.-L., Upadhyayula S., Milkie D. E., Singh V., Wang K., Swinburne I. A., Mosaliganti K. R., Collins Z. M., Hiscock T. W., Shea J., Kohrman A. Q., Medwig T. N., Dambournet D., Forster R., Cunniff B., Ruan Y., Yashiro H., Scholpp S., Meyerowitz E. M., Hockemeyer D., Drubin D. G., Martin B. L., Matus D. Q., Koyama M., Megason S. G., Kirchhausen T., Betzig E. (2018). Observing the Cell
in Its Native State: Imaging Subcellular Dynamics in Multicellular
Organisms. Science.

[20] Daugird T. A., Shi Y., Holland K. L., Rostamian H., Liu Z., Lavis L. D., Rodriguez J., Strahl B. D., Legant W. R. (2024). Correlative Single
Molecule Lattice Light Sheet Imaging Reveals the Dynamic Relationship
between Nucleosomes and the Local Chromatin Environment. Nat. Commun..

[21] Bishop K. W., Glaser A. K., Liu J. T. C. (2020). Performance Tradeoffs
for Single-
and Dual-Objective Open-Top Light-Sheet Microscope Designs: A Simulation-Based
Analysis. Biomed. Opt. Express.

[22] van
Niekerk D. D., Gustavsson A.-K., Mojica-Benavides M., Adiels C. B., Goksör M., Snoep J. L. (2019). Phosphofructokinase
Controls the Acetaldehyde-Induced Phase Shift in Isolated Yeast Glycolytic
Oscillators. Biochem. J..

[23] Gustavsson A.-K., Adiels C. B., Mehlig B., Goksör M. (2015). Entrainment
of Heterogeneous Glycolytic Oscillations in Single Cells. Sci. Rep.

[24] Gustavsson A.-K., van Niekerk D. D., Adiels C. B., Kooi B., Goksör M., Snoep J. L. (2014). Allosteric Regulation of Phosphofructokinase Controls
the Emergence of Glycolytic Oscillations in Isolated Yeast Cells. FEBS J..

[25] Gustavsson A.-K., van Niekerk D. D., Adiels C. B., Goksör M., Snoep J. L. (2014). Heterogeneity of
Glycolytic Oscillatory Behaviour in
Individual Yeast Cells. FEBS Lett..

[26] Gustavsson A.-K., van Niekerk D. D., Adiels C. B., du Preez F. B., Goksör M., Snoep J. L. (2012). Sustained Glycolytic Oscillations in Individual Isolated
Yeast Cells. FEBS J..

[27] Moore R. P., O’Shaughnessy E. C., Shi Y., Nogueira A. T., Heath K. M., Hahn K. M., Legant W. R. (2021). A Multi-Functional
Microfluidic Device Compatible with Widefield and Light Sheet Microscopy. Lab Chip.

[28] Zhang J., Zhang M., Wang Y., Donarski E., Gahlmann A. (2021). Optically
Accessible Microfluidic Flow Channels for Noninvasive High-Resolution
Biofilm Imaging Using Lattice Light Sheet Microscopy. J. Phys. Chem. B.

[29] Fan Y.-J., Hsieh H.-Y., Tsai S.-F., Wu C.-H., Lee C.-M., Liu Y.-T., Lu C.-H., Chang S.-W., Chen B.-C. (2021). Microfluidic
Channel Integrated with a Lattice Lightsheet Microscopic System for
Continuous Cell Imaging. Lab Chip.

[30] Han X., Su Y., White H., O’Neill K. M., Morgan N. Y., Christensen R., Potarazu D., Vishwasrao H. D., Xu S., Sun Y., Huang S., Moyle M. W., Dai Q., Pommier Y., Giniger E., Albrecht D. R., Probst R., Shroff H. (2021). A Polymer
Index-Matched to Water Enables Diverse Applications in Fluorescence
Microscopy. Lab Chip.

[31] Shi Y., Daugird T. A., Legant W. R. (2024). Posterior
Approach to Correct for
Focal Plane Offsets in Lattice Light-Sheet Structured Illumination
Microscopy. J. Biomed. Opt..

[32] Galland R., Grenci G., Aravind A., Viasnoff V., Studer V., Sibarita J.-B. (2015). 3D High- and Super-Resolution
Imaging Using Single-Objective
SPIM. Nat. Methods.

[33] Saliba N., Gagliano G., Gustavsson A.-K. (2024). Whole-Cell
Multi-Target Single-Molecule
Super-Resolution Imaging in 3D with Microfluidics and a Single-Objective
Tilted Light Sheet. Nat. Commun..

[34] Tokunaga M., Imamoto N., Sakata-Sogawa K. (2008). Highly Inclined
Thin Illumination
Enables Clear Single-Molecule Imaging in Cells. Nat. Methods.

[35] Tang J., Han K. Y. (2018). Extended Field-of-View Single-Molecule Imaging by Highly
Inclined Swept Illumination. Optica.

[36] Kasu R., Luo H., Luckhart S., Christodoulides D. N., Vasdekis A. E. (2025). Single-Objective
Airy Light-Sheet Imaging. ACS Photonics.

[37] Meddens M. B. M., Liu S., Finnegan P. S., Edwards T. L., James C. D., Lidke K. A. (2016). Single Objective Light-Sheet Microscopy for High-Speed
Whole-Cell 3D Super-Resolution. Biomed. Opt.
Express, BOE.

[38] Pavani S. R. P., Piestun R. (2008). High-Efficiency Rotating Point Spread Functions. Opt. Express, OE.

[39] Pavani S. R. P., Thompson M. A., Biteen J. S., Lord S. J., Liu N., Twieg R. J., Piestun R., Moerner W. E. (2009). Three-Dimensional,
Single-Molecule Fluorescence Imaging beyond the Diffraction Limit
by Using a Double-Helix Point Spread Function. Proc. Natl. Acad. Sci. U. S. A..

[40] Bennett H. W., Gustavsson A.-K., Bayas C. A., Petrov P. N., Mooney N., Moerner W. E., Jackson P. K. (2020). Novel Fibrillar Structure in the
Inversin Compartment of Primary Cilia Revealed by 3D Single-Molecule
Superresolution Microscopy. Mol. Biol. Cell.

[41] Weiss, L. E. ; Love, J. F. ; Yoon, J. ; Comerci, C. J. ; Milenkovic, L. ; Kanie, T. ; Jackson, P. K. ; Stearns, T. ; Gustavsson, A.-K. Chapter 4 - Single-Molecule Imaging in the Primary Cilium. In Methods in Cell Biology; Bravo-San Pedro, J. M. ; Galluzzi, L. , Eds.; From Mechanisms to Disease - Part B; Academic Press: Cilia, 2023; Vol. 176, pp 59–83 10.1016/bs.mcb.2023.01.003. PMC10509820 37164543

[42] Nakatani Y., Gaumer S., Shechtman Y., Gustavsson A.-K. (2024). Long-Axial-Range
Double-Helix Point Spread Functions for 3D Volumetric Super-Resolution
Imaging. J. Phys. Chem. B.

[43] Kanie T., Liu B., Love J. F., Fisher S. D., Gustavsson A.-K., Jackson P. K. (2025). A Hierarchical Pathway
for Assembly of the Distal Appendages
That Organize Primary Cilia. eLife.

[44] Möckl L., Pedram K., Roy A. R., Krishnan V., Gustavsson A.-K., Dorigo O., Bertozzi C. R., Moerner W. E. (2019). Quantitative Super-Resolution
Microscopy of the Mammalian Glycocalyx. Dev.
Cell.

[45] Speiser A., Müller L.-R., Hoess P., Matti U., Obara C. J., Legant W. R., Kreshuk A., Macke J. H., Ries J., Turaga S. C. (2021). Deep Learning
Enables Fast and Dense Single-Molecule
Localization with High Accuracy. Nat. Methods.

[46] Shi Y., Daugird T. A., Legant W. R. (2022). A Quantitative Analysis of Various
Patterns Applied in Lattice Light Sheet Microscopy. Nat. Commun..

[47] Brzobohatý O., Čižmár T., Zemánek P. (2008). High Quality
Quasi-Bessel Beam Generated by Round-Tip Axicon. Opt. Express.

[48] Remacha E., Friedrich L., Vermot J., Fahrbach F. O. (2020). How to Define and
Optimize Axial Resolution in Light-Sheet Microscopy: A Simulation-Based
Approach. Biomed. Opt. Express.

[49] Chang B.-J., Dean K. M., Fiolka R. (2020). Systematic
and Quantitative Comparison
of Lattice and Gaussian Light-Sheets. Opt. Express.

[50] Stockhausen A., Bürgers J., Rodriguez-Gatica J. E., Schweihoff J., Merkel R., Prigge J. M., Schwarz M. K., Kubitscheck U. (2020). Hard-Wired
Lattice Light-Sheet Microscopy for Imaging of Expanded Samples. Opt. Express.

[51] Nelson T., Vargas-Hernández S., Freire M., Cheng S., Gustavsson A.-K. (2024). Multimodal
Illumination Platform for 3D Single-Molecule
Super-Resolution Imaging throughout Mammalian Cells. Biomed. Opt. Express.

[52] Gustavsson A.-K., Petrov P. N., Lee M. Y., Shechtman Y., Moerner W. E. (2018). 3D Single-Molecule Super-Resolution
Microscopy with
a Tilted Light Sheet. Nat. Commun..

[53] Bouchal Z., Wagner J., Chlup M. (1998). Self-Reconstruction
of a Distorted
Nondiffracting Beam. Opt. Commun..

[54] Fahrbach F. O., Simon P., Rohrbach A. (2010). Microscopy with Self-Reconstructing
Beams. Nat. Photonics.

[55] Gong L., Ren Y.-X., Xue G.-S., Wang Q.-C., Zhou J.-H., Zhong M.-C., Wang Z.-Q., Li Y.-M. (2013). Generation of Nondiffracting
Bessel Beam Using Digital Micromirror Device. Appl. Opt..

[56] Broky J., Siviloglou G. A., Dogariu A., Christodoulides D. N. (2008). Self-Healing
Properties of Optical Airy Beams. Opt. Express.

[57] Hüpfel M., Nienhaus G. U. (2024). Beam Shaping in Light-Sheet Microscopy: An Experimental
Analysis. J. Phys. Photonics.

[58] Shi F., Wen J., Lei D. (2020). High-Efficiency,
Large-Area Lattice Light-Sheet Generation
by Dielectric Metasurfaces. Nanophotonics.

[59] Shi Y., Tabet J. S., Milkie D. E., Daugird T. A., Yang C. Q., Ritter A. T., Giovannucci A., Legant W. R. (2024). Smart Lattice Light-Sheet
Microscopy for Imaging Rare and Complex Cellular Events. Nat. Methods.

[60] Zhang X., Flores L. R., Keeling M. C., Sliogeryte K., Gavara N. (2020). Ezrin Phosphorylation at T567 Modulates Cell Migration,
Mechanical Properties, and Cytoskeletal Organization. Int. J. Mol. Sci..

[61] Chowdhury, P. ; Wang, X. ; Love, J. F. ; Vargas-Hernandez, S. ; Nakatani, Y. ; Grimm, S. L. ; Mezquita, D. ; Mason, F. M. ; Martinez, E. D. ; Coarfa, C. ; Walker, C. L. ; Gustavsson, A.-K. ; Dere, R. Lysine Demethylase 4A Is a Centrosome Associated Protein Required for Centrosome Integrity and Genomic Stability bioRxiv 2024, p 2024.02.20.581246 10.1101/2024.02.20.581246. PMC12820605 40875546

[62] Stahley S. N., Bartle E. I., Atkinson C. E., Kowalczyk A. P., Mattheyses A. L. (2016). Molecular Organization of the Desmosome as Revealed
by Direct Stochastic Optical Reconstruction Microscopy. J. Cell Sci..

[63] Bharathan N. K., Mattheyses A. L., Kowalczyk A. P. (2024). The Desmosome Comes into Focus. J. Cell Biol..

[64] Gao L., Shao L., Chen B.-C., Betzig E. (2014). 3D Live Fluorescence
Imaging of Cellular Dynamics Using Bessel Beam Plane Illumination
Microscopy. Nat. Protoc..

[65] Gebhardt J. C. M., Suter D. M., Roy R., Zhao Z. W., Chapman A. R., Basu S., Maniatis T., Xie X. S. (2013). Single-Molecule
Imaging of Transcription Factor Binding to DNA in Live Mammalian Cells. Nat. Methods.

[66] Nozaki T., Imai R., Tanbo M., Nagashima R., Tamura S., Tani T., Joti Y., Tomita M., Hibino K., Kanemaki M. T., Wendt K. S., Okada Y., Nagai T., Maeshima K. (2017). Dynamic Organization of Chromatin
Domains Revealed by Super-Resolution Live-Cell Imaging. Mol. Cell.

[67] Gustavsson A.-K., Ghosh R. P., Petrov P. N., Liphardt J. T., Moerner W. E. (2022). Fast and
Parallel Nanoscale Three-Dimensional Tracking of Heterogeneous Mammalian
Chromatin Dynamics. Mol. Biol. Cell.

[68] Lu Y., Parker Kh., Wang W. (2006). Effects of
Osmotic Pressure in the
Extracellular Matrix on Tissue Deformation. Philos. Trans. R. Soc., A.

[69] Shechtman Y., Gustavsson A.-K., Petrov P. N., Dultz E., Lee M. Y., Weis K., Moerner W. E. (2017). Observation of Live Chromatin Dynamics
in Cells via 3D Localization Microscopy Using Tetrapod Point Spread
Functions. Biomed. Opt. Express.

[70] Gustavsson A.-K., Banaeiyan A. A., van Niekerk D. D., Snoep J. L., Adiels C. B., Goksör M. (2019). Studying Glycolytic Oscillations in Individual Yeast
Cells by Combining Fluorescence Microscopy with Microfluidics and
Optical Tweezers. Curr. Prot. Cell Biol..

[71] Park H., Sut T. N., Yoon B. K., Zhdanov V. P., Kim J. W., Cho N.-J., Jackman J. A. (2022). Multivalency-Induced
Shape Deformation
of Nanoscale Lipid Vesicles: Size-Dependent Membrane Bending Effects. J. Phys. Chem. Lett..

[72] Deviri D., Safran S. A. (2022). Balance of Osmotic
Pressures Determines the Nuclear-to-Cytoplasmic
Volume Ratio of the Cell. Proc. Natl. Acad.
Sci. U. S. A..

[73] Cheng J., Allgeyer E. S., Richens J. H., Dzafic E., Palandri A., Lewków B., Sirinakis G., St Johnston D. (2021). A Single-Molecule
Localization Microscopy Method for Tissues Reveals Nonrandom Nuclear
Pore Distribution in Drosophila. J. Cell Sci..

[74] Cabillic M., Forriere H., Bettarel L., Butler C., Neuhaus A., Idrissi I., Sambrano-Lopez M. E., Rossbroich J., Müller L.-R., Ries J., Grenci G., Viasnoff V., Levet F., Sibarita J.-B., Galland R. (2025). In-Depth Single Molecule
Localization Microscopy Using Adaptive Optics and Single Objective
Light-Sheet Microscopy. Nat. Commun..

[75] Hu Q., Hailstone M., Wang J., Wincott M., Stoychev D., Atilgan H., Gala D., Chaiamarit T., Parton R. M., Antonello J., Packer A. M., Davis I., Booth M. J. (2023). Universal Adaptive
Optics for Microscopy through Embedded
Neural Network Control. Light Sci. Appl..

[76] Huisken J., Stainier D. Y. R. (2007). Even Fluorescence Excitation by Multidirectional Selective
Plane Illumination Microscopy (mSPIM). Opt.
Lett..

[77] Ovesný M., Křížek P., Borkovec J., Švindrych Z., Hagen G. M. (2014). ThunderSTORM: A Comprehensive ImageJ Plug-in for PALM
and STORM Data Analysis and Super-Resolution Imaging. Bioinformatics.

[78] Quan T., Li P., Long F., Zeng S., Luo Q., Hedde P. N., Nienhaus G. U., Huang Z.-L. (2010). Ultra-Fast, High-Precision Image
Analysis for Localization-Based Super Resolution Microscopy. Opt. Express, OE.

[79] Easy-DHPSF 2.0: open-source software for three-dimensional localization and two-color registration of single molecules with nanoscale accuracy | Protocol Exchange, https://protocolexchange.researchsquare.com/article/pex-129/v1 (accessed Feb 20, 2025).

[80] Stallinga, S. ; Rieger, B. The Effect of Background on Localization Uncertainty in Single Emitter Imaging, 2012 9th IEEE International Symposium on Biomedical Imaging (ISBI); 2012; pp 988–991 10.1109/ISBI.2012.6235723.

[81] Huang B., Wang W., Bates M., Zhuang X. (2008). Three-Dimensional
Super-Resolution
Imaging by Stochastic Optical Reconstruction Microscopy. Science.

[82] Cole R. W., Jinadasa T., Brown C. M. (2011). Measuring
and Interpreting Point
Spread Functions to Determine Confocal Microscope Resolution and Ensure
Quality Control. Nat. Protoc..

[83] Petrov P. N., Moerner W. E. (2020). Addressing Systematic Errors in Axial Distance Measurements
in Single-Emitter Localization Microscopy. Opt.
Express.

