# Supplementary material for: Single-Objective Lattice Light Sheet Microscopy with Microfluidics for Single-Molecule Super-Resolution Imaging of Mammalian Cells
Source: ACS Photonics. 2025 Dec 12;13(1):249–62. doi: 10.1021/acsphotonics.5c02201 (PMC12784397; doi:10.1021/acsphotonics.5c02201)
Supplement: Supplementary file 1 [file ph5c02201_si_001.pdf]

Supporting Information for Single-objective Lattice Light Sheet Microscopy with Microfluidics for Single-molecule Super-resolution Imaging of Mammalian Cells

Siyang Cheng[1,2,3], Nahima Saliba[1], Gabriella Gagliano[1,2,3], Prakash Joshi[1], Anna-Karin Gustavsson[1,2,4,5,6,7,]*

[1]Department of Chemistry, Rice University, Houston, Texas 77005, United States
[2]Smalley-Curl Institute, Rice University, Houston, Texas 77005, United States
[3]Applied Physics Program, Rice University, Houston, Texas 77005, United States
[4]Department of BioSciences, Rice University, Houston, Texas 77005, United States
[5]Department of Electrical and Computer Engineering, Rice University, Houston, Texas 77005, United States
[6]Center for Nanoscale Imaging Sciences, Rice University, Houston, Texas 77005, United States
[7]Department of Cancer Biology, University of Texas MD Anderson Cancer Center, Houston, Texas 77005, United States
**anna-karin.gustavsson@rice.edu*

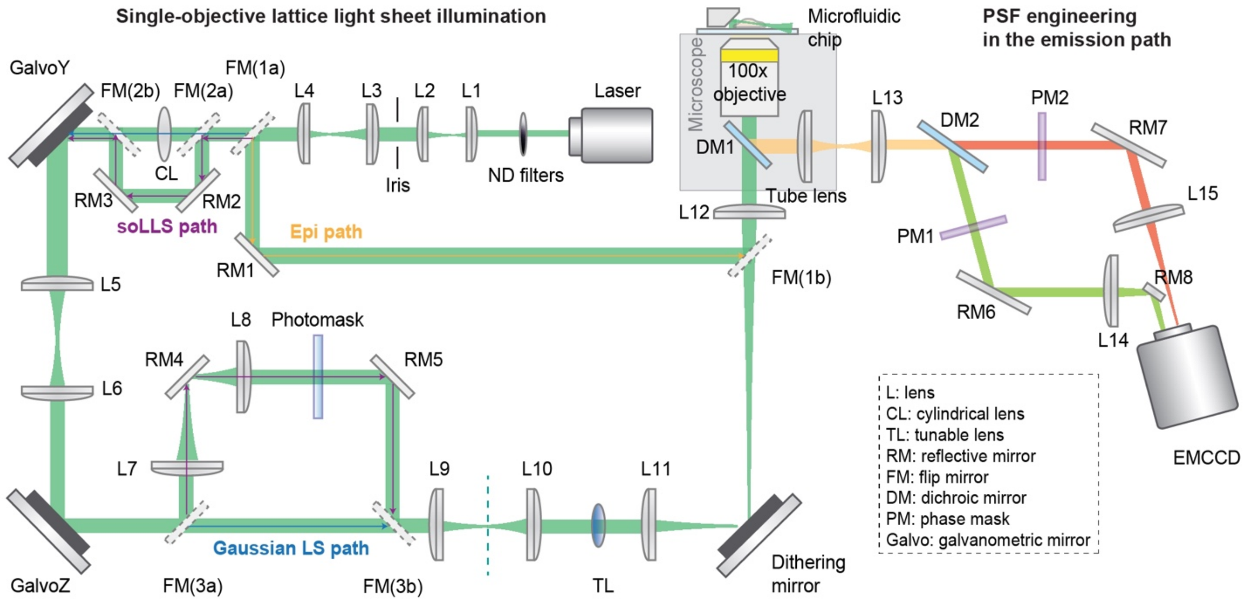

Fig. S1. Simplified schematic of the optical setup. The single-objective lattice light sheet (soLLS) setup is coaligned with a single-objective Gaussian light sheet (LS) path and a widefield epi-illumination path. FM: flip mirrors that allow for switching between the three illumination modalities. When FM(1a) and FM(1b) are flipped up, the setup works in epi-illumination mode. When FM(1a) and FM(1b) are flipped down and FM(2a), FM(2b), FM(3a), and FM(3b) are flipped up, the laser beam is shaped by the photomask to generate a LLS, and the setup works in soLLS illumination mode. When all the flip mirrors are flipped down, the laser beam is shaped by the cylindrical lens, and the setup works in single-objective Gaussian LS illumination mode. An iris is used for tuning the thickness of the Gaussian LS. GalvoZ, GalvoY, and TL: galvanometric mirrors and a tunable lens for LS steering. GalvoY is also used for scanning along the width of the LLS to homogenize its profile. These components are shared between the Gaussian LS path and the LLS path. Dithering mirror: mirror used to dither (adjust the angle of) the LS in the sample plane. DM: dichroic mirror. PM: phase masks in the emission paths for PSF engineering. The schematic is not to scale. For clarity, not all lenses used to adjust the collimated beam size are shown in the simplified schematic. A list of all components with part numbers is provided in Table S2. Filters, polarizer, and quarter-wave plate are not shown in the schematic but are included in Table S2.

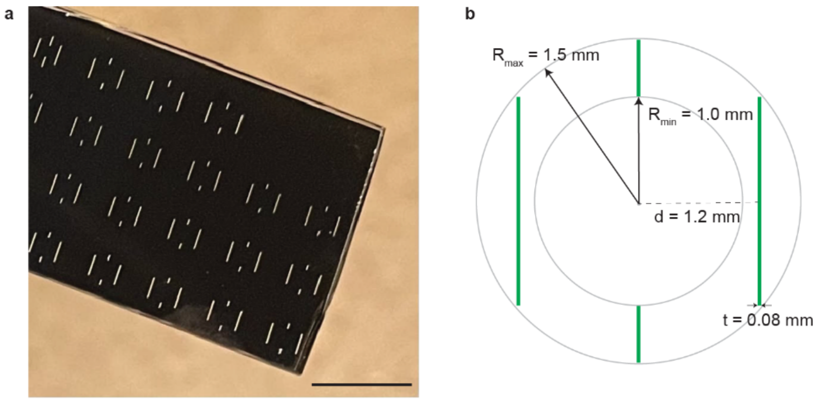

Fig. S2. Photograph and schematic of the photomask used in the soLLS platform. a, A custom-designed photomask was implemented to generate the LLS. The print incorporates multiple mask pattern designs to facilitate flexible alignment adjustments. Scale bar: 1 cm. **b,** Dimensions of the slits pattern used in this work.

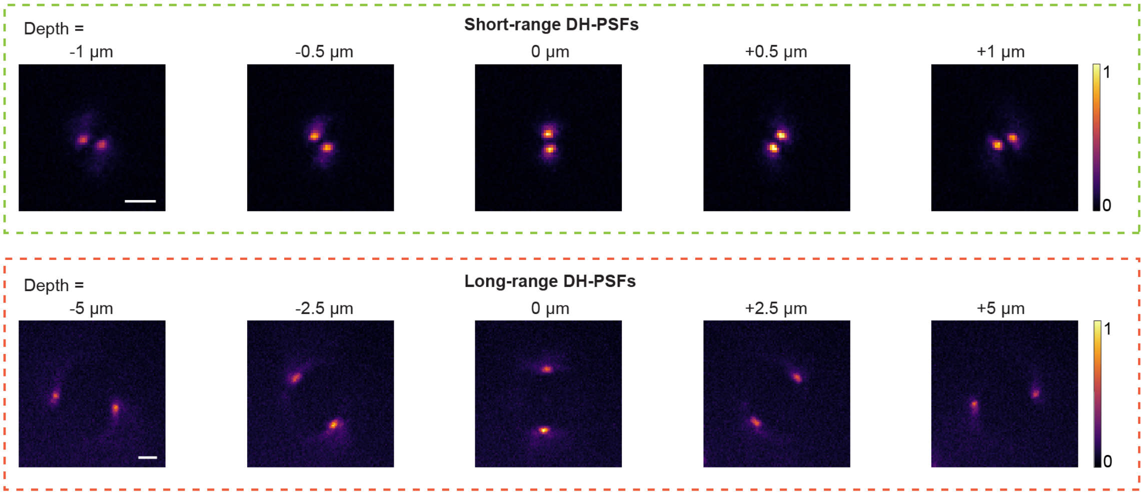

Fig. S3. Point spread function (PSF) engineering of the emission light yields 3D information. In this work, short axial range double-helix (DH)-PSFs (top row), which have an experimental effective axial range of ~3 μm, was implemented for 3D localization of the single-molecule data. Long axial range DH-PSFs (bottom row), which have an experimental effective axial range of ~12 μm, were used for localizing fiducial beads for drift correction. Scale bars: 2 μm. The colorbars show normalized intensity.

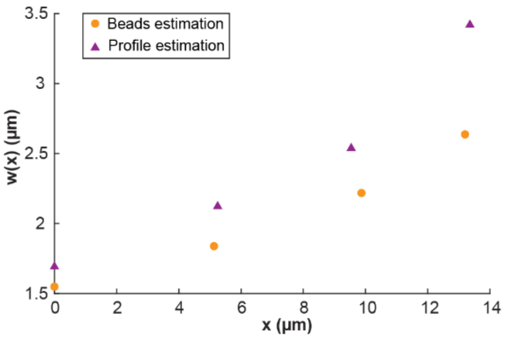

Fig. S4. Estimation of the thickness of the soLLS from intensity measurement of fluorescent beads. Fluorescent beads were immobilized in 1% (w/v) agarose in a microfluidic chip with a mirror at 45°. The sample was scanned in 1D across the thin end (side view) of the soLLS. The thickness ($1/e^2$ radius) of the soLLS was estimated by fitting the intensity plot of the beads with a Gaussian function. The results show that the estimated beam thickness of the soLLS is smaller than the estimation from the projected profile captured using a CF568 solution in a microfluidic chip (Fig. 2c,f), as expected. Importantly, the trend of beam thickness during propagation remains consistent in both measurements.

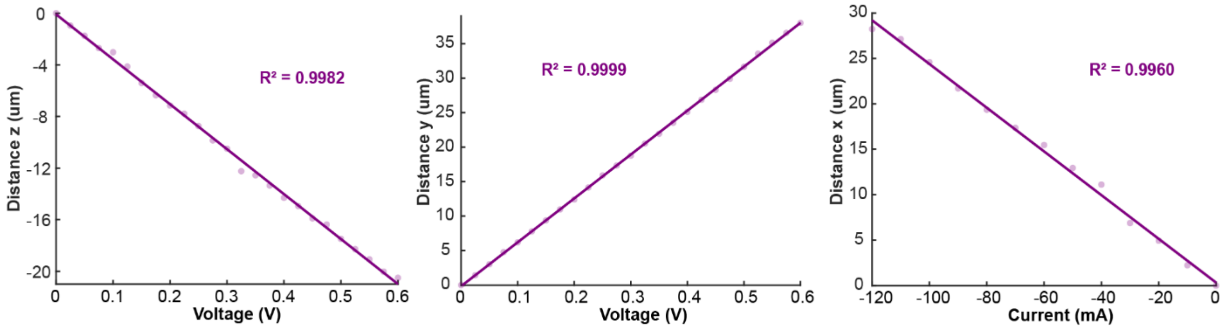

Fig. S5. Experimental calibration of the beam steering units in the soLLS setup. The beam steering units featuring two galvanometric mirrors (GalvoZ and GalvoY) and a tunable lens (TL) to enable linear repositioning of the soLLS in three dimensions, where the LLS dimensions are decoupled from the steering. Experimentally-derived calibrations show that every 0.01 V applied to galvo Z/Y results in a translation in the z/y direction (up and down for sectioning and within the light sheet plane, respectively) of approximately 0.35/0.64 µm. The tunable lens shifts the light sheet focus 2.41 µm in the x direction (along the beam propagation) for every current step of 10 mA to enable sectioning of cells at various distances from the side wall.

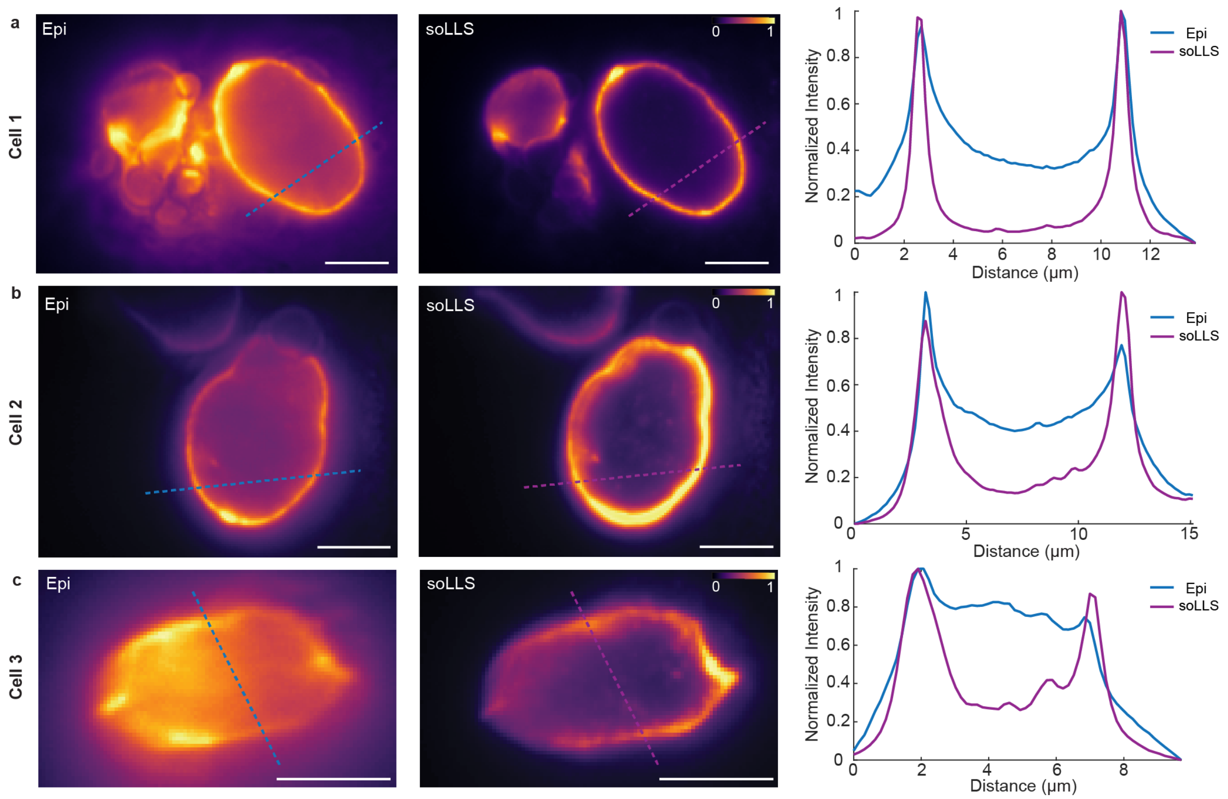

Fig. S6. Technical replicates demonstrating reproducibility of the diffraction-limited lamin B1 imaging shown in Figure 4a. a-c, Diffraction-limited images of lamin B1 in three different U2OS cells acquired with widefield epi- (Epi) or soLLS illumination. Graphs show the comparison of normalized intensity distributions across line scans in the same cell under epi- or soLLS illumination, demonstrating reproducible signal-to-background ratio (SBR) improvement with soLLS illumination compared to widefield epi-illumination. SBR improvements were 7.6-fold, 3.0-fold, and 3.1-fold in **a**, **b**, and **c**, respectively. Scale bars: 5 μm. The colorbars show normalized intensity.

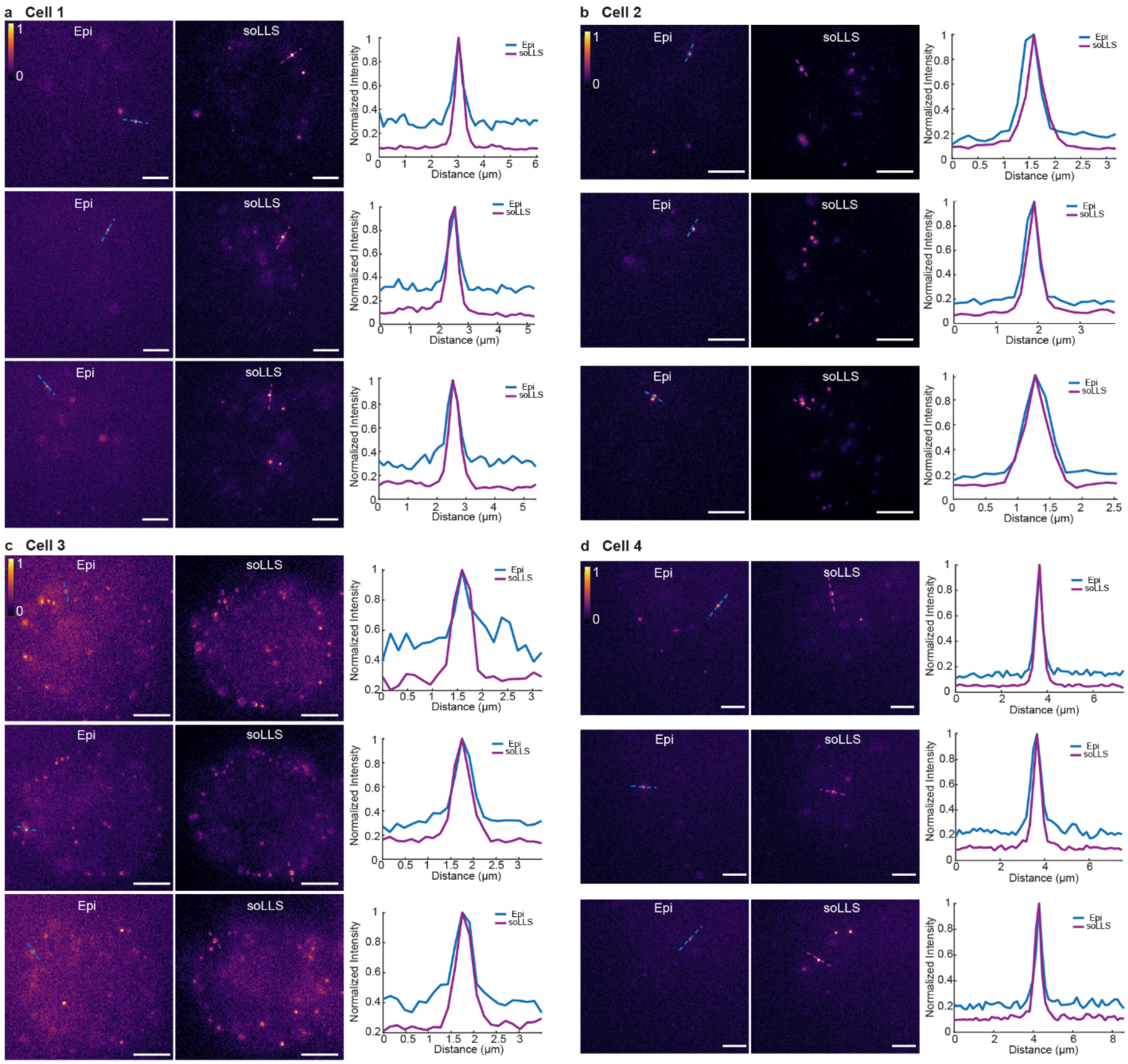

Fig. S7. Technical replicates demonstrating reproducibility of 2D single-molecule imaging of lamin B1 shown in Figure 4b. a-d, 2D single-molecule images of DNA-PAINT labeled lamin B1 in four different U2OS cells acquired with widefield epi- (Epi) or soLLS illumination. Graphs show the comparison of normalized intensity distributions across line scans of the emitters under epi- or soLLS illumination. SBR improvements were 3.9±0.4-fold, 1.9±0.3-fold, 1.9±0.2-fold, and 2.5±0.5-fold in **a**, **b**, **c**, and **d**, respectively (mean ± standard deviation, n = 3 molecules per cell). Scale bars: 5 µm. The colorbars show normalized intensity.

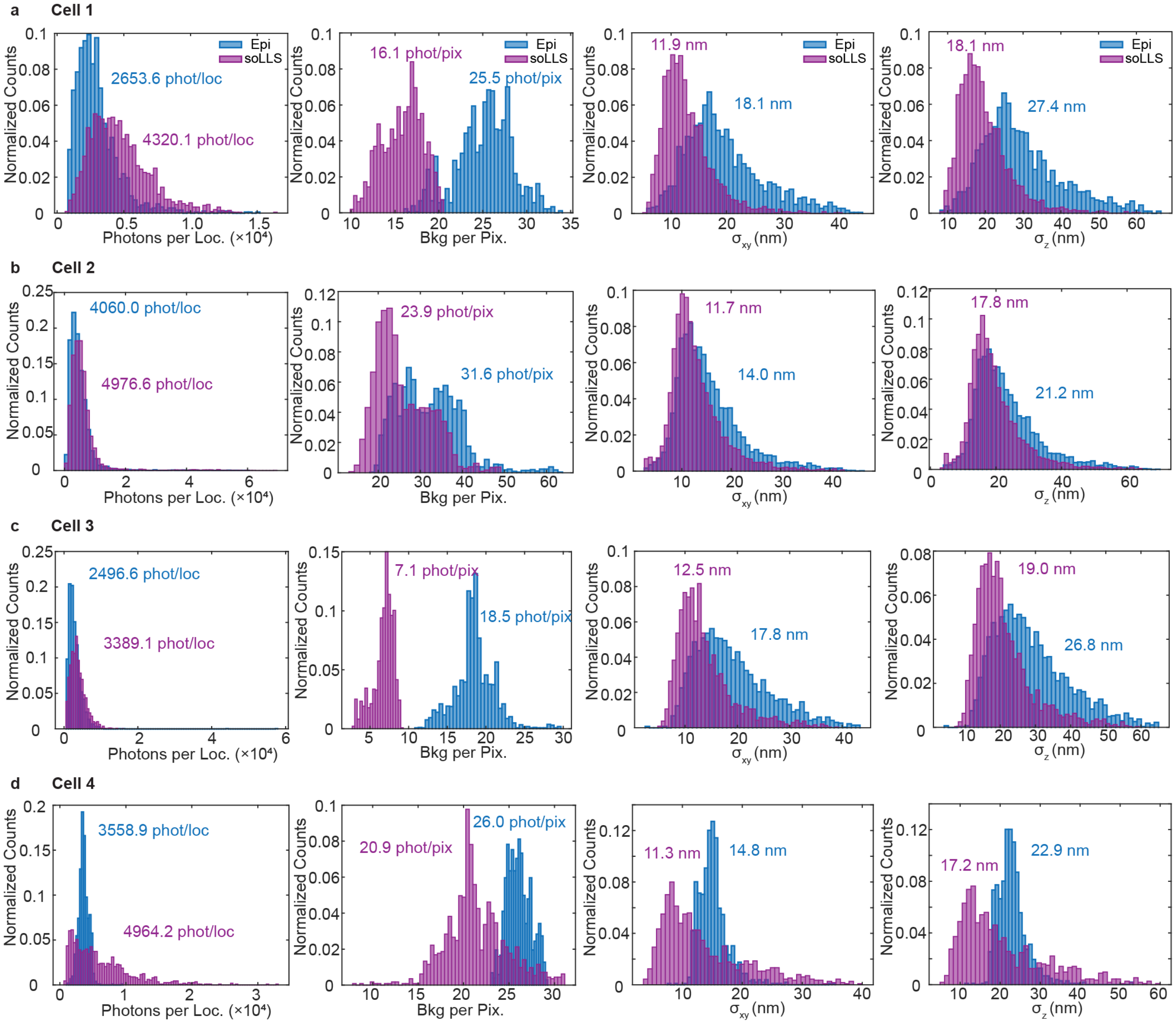

Fig. S8. Technical replicates demonstrating reproducibility of 3D single-molecule super-resolution imaging of lamin B1 shown in Figure 4c. a-d, Histograms showing comparisons of signal photons per localization, background photons per pixel, lateral (xy) localization precision, and axial (z) localization precision of the localized emitters in the same cell under epi- (Epi) or soLLS illumination for four different cells. Median values of signal photons per localization, background photons per pixel, and localization precisions are indicated in each panel. Background reduction and improvements in localization precisions were demonstrated for each cell.

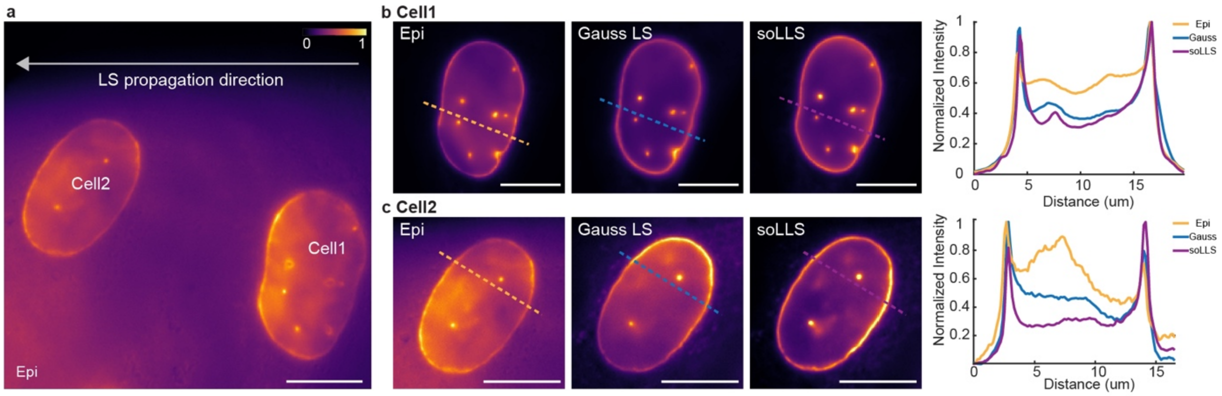

Fig. S9. Direct comparison between soLLS and Gaussian LS in diffraction-limited imaging of lamin B1. a, A whole field of view showing two cells acquired with epi-illumination. **b,c,** Images and line scans show that **b,** both LSs provide substantial signal-to-background (SBR) improvement over epi-illumination in the first cell sectioned by the LSs, but **c,** the soLLS outperforms the Gaussian LS for sectioning of the second cell by providing more uniform illumination and a 1.9-fold improvement in SBR due to its superior propagation properties. Scale bars: 10 µm. The colorbar shows normalized intensity. The intensity is normalized independently for each image. In **a** and **c**, the chip contained 1 µM Cy3B imager solution.

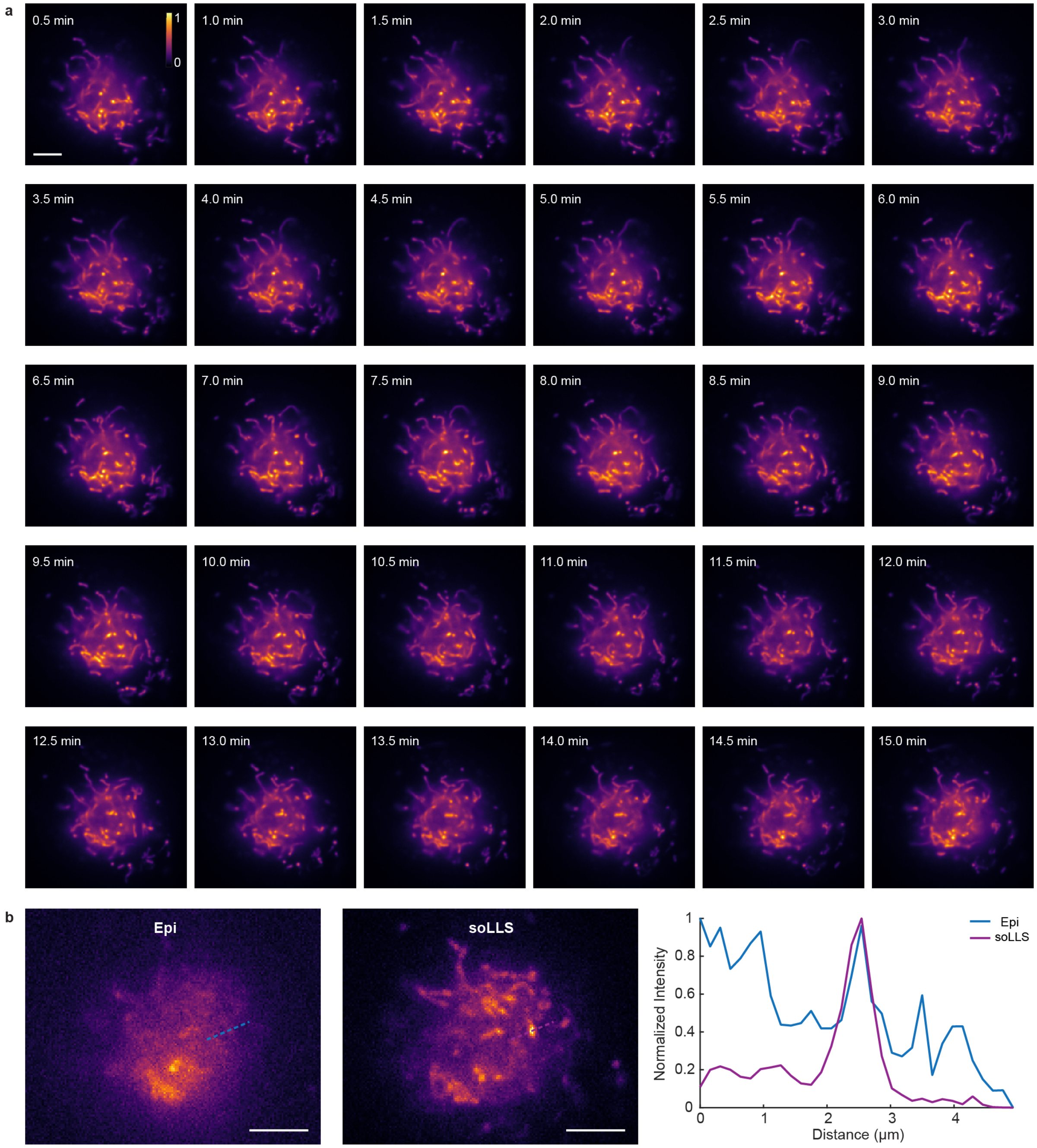

Fig. S10. Live-cell imaging with soLLS. a, Mitochondria in a live U2OS cell imaged with soLLS. Image stacks of 50 frames were acquired and averaged for every 30 seconds over a 15-minute period. **b,** Single image frames and line scans of the same cell demonstrating the SBR improvement with soLLS compared with epi-illumination in live-cell imaging. Scale bars: 5 μm. The colorbar shows normalized intensity. The intensity is normalized independently for each image.

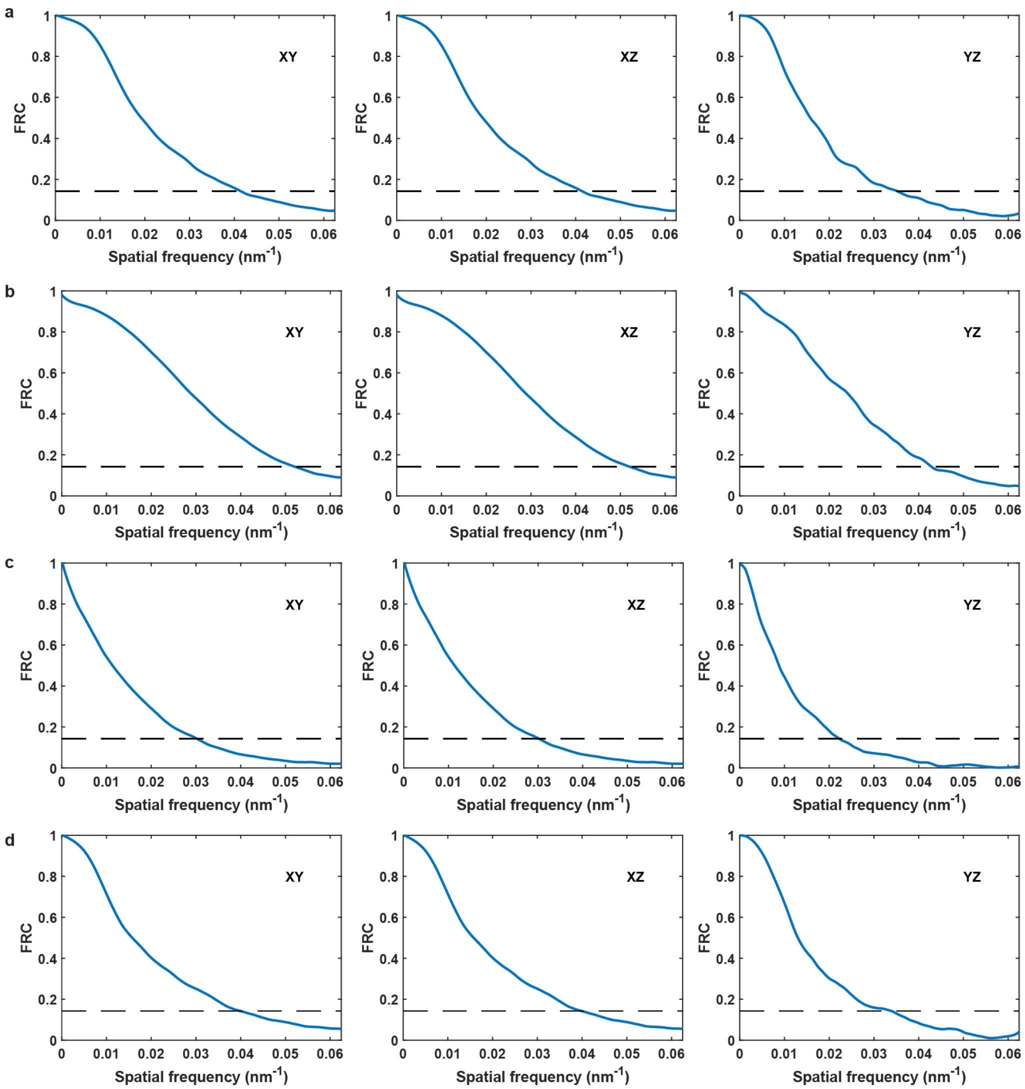

Fig. S11. Fourier ring correlation (FRC) analysis for the 3D super-resolution reconstructions shown in Fig. 5. **a-b,** FRC analysis of the TOMM20 and LAP2 reconstructions shown in Fig. 5b-c, respectively. The resulting FRC resolutions in the xy/xz/yz planes were found to be **a,** 24.2/30.8/28.5 nm for TOMM20 and **b,** 19.2/23.4/23.4 nm for LAP2. **c-d,** FRC analysis of the lamin A/C and TOMM20 reconstructions shown in Fig. 5f-g, respectively. The resulting FRC resolutions in the xy/xz/yz planes were found to be **c,** 33.1/45.1/45.0 nm for lamin A/C and **d,** 25.1/33.6/29.5 nm for TOMM20. All FRC curves were calculated using a super-resolution pixel size of 8 nm in Vutara SRX.

Table S1. Absolute numbers of localizations in each region shown in Fig. 3. The data is shown as mean ± standard deviation for n = 9 fields of view each for the two indicated illumination methods.

	soLLS	Gaussian LS
Region 1	44488 ± 7820	49442 ± 13340
Region 2	31426 ± 7444	12829 ± 8964
Region 3	27087 ± 7861	2427 ± 2980
Region 4	17989 ± 6802	746 ± 1341
Region 5	7754 ± 4675	21 ± 22

Table S2. Components list of the optical setup.

Label	Description	Part number
**Excitation optics**		
Laser	560 nm	2RU-VFL-P-1000-560-FC, MPB
/	Laser clean-up filter	FF01-554/23-25, Semrock
/	Linear polarizer	LPVISC050MP2, Thorlabs
/	Quarter wave-plate	Z-10-A.250-B-556, Tower Optical
ND filters	Neutral density filters	NE01B - NE50B series, Thorlabs
**Lenses and mirrors**		
L1	f = 25 mm	LA1951-A, Thorlabs
L2	f = 150 mm	LA1417-A, Thorlabs
L3, L5, L9	f = 150 mm	AC254-150-A-ML, Thorlabs
L4, L6, L10	f = 75 mm	AC254-075-A-ML, Thorlabs
L7	f = 200 mm	AC254-200-A, Thorlabs
L8	f = 100 mm	AC254-100-A, Thorlabs
L11, L12	f = 300 mm	AC508-300-A, Thorlabs
L13, L14, L15	f = 80 mm	AC508-080-AB, Thorlabs
FM	Flip-mounted mirrors	TRF290, Thorlabs
RM1	2" reflective mirrors	BB2-E02, Thorlabs
RM2 – RM7	1" reflective mirrors	BB1-E02, Thorlabs
RM8	D-shaped mirror	BBD1-E02, Thorlabs
**Beam steering**		
Galvo	Galvanometric mirrors	GVS011, Thorlabs
TL	Tunable lens	EL-3-10-VIS-26D-FPC, Optotune
Dithering mirror	Dithering mirror	SP30Y-AG, Thorlabs
**Beam shaping**		
Photomask	Photomask for generating LLS	Custom-designed, HTA photomask
CL	Cylindrical lens, f = 150 mm	ACY254-150-A, Thorlabs
Iris	Iris for tuning the Gaussian LS	IDA25, Thorlabs
PM1	Short-range DH phase mask	DH1-580, Double Helix Optics
PM2	Long-range DH phase mask	DH12R-670, Double Helix Optics
**Microscopes**		
Microscope	Inverted microscope	IX83, Olympus
100x objective	100x, NA 1.45	UPLXAPO100X, Olympus
**Dichroic mirrors, emission filters**		
DM1	Quad-edge dichroic mirror	ZT405/488/561/ 640rpcV3, Chroma
DM2	Dichroic mirror	T660lpxr-UF3, Chroma
/	Notch filters	ZET642nf, ZET561nf, Chroma
/	Bandpass filters	ET650/70m, ET700/75m, Chroma
**Camera**		
EMCCD	EMCCD camera	iXon Ultra 897, Andor
